# Deep representation learning identifies associations between physical activity and sleep patterns during pregnancy and prematurity

**DOI:** 10.1038/s41746-023-00911-x

**Published:** 2023-09-28

**Authors:** Neal G. Ravindra, Camilo Espinosa, Eloïse Berson, Thanaphong Phongpreecha, Peinan Zhao, Martin Becker, Alan L. Chang, Sayane Shome, Ivana Marić, Davide De Francesco, Samson Mataraso, Geetha Saarunya, Melan Thuraiappah, Lei Xue, Brice Gaudillière, Martin S. Angst, Gary M. Shaw, Erik D. Herzog, David K. Stevenson, Sarah K. England, Nima Aghaeepour

**Affiliations:** 1 Department of Anesthesiology, Perioperative and Pain Medicine, Stanford School of Medicine, Stanford, CA USA; 2 Department of Pediatrics, Stanford School of Medicine, Stanford, CA USA; 3 Department of Biomedical Data Science, Stanford University, Stanford, CA USA; 4 Department of Pathology, Stanford School of Medicine, Stanford, CA USA; 5 Department of Biology, Washington University in St. Louis, St. Louis, MO USA; 6 Department of Obstetrics and Gynecology, Washington University in St. Louis, St. Louis, MO USA

**Keywords:** Computer science, Preterm birth

## Abstract

Preterm birth (PTB) is the leading cause of infant mortality globally. Research has focused on developing predictive models for PTB without prioritizing cost-effective interventions. Physical activity and sleep present unique opportunities for interventions in low- and middle-income populations (LMICs). However, objective measurement of physical activity and sleep remains challenging and self-reported metrics suffer from low-resolution and accuracy. In this study, we use physical activity data collected using a wearable device comprising over 181,944 h of data across *N* = 1083 patients. Using a new state-of-the art deep learning time-series classification architecture, we develop a ‘clock’ of healthy dynamics during pregnancy by using gestational age (GA) as a surrogate for progression of pregnancy. We also develop novel interpretability algorithms that integrate unsupervised clustering, model error analysis, feature attribution, and automated actigraphy analysis, allowing for model interpretation with respect to sleep, activity, and clinical variables. Our model performs significantly better than 7 other machine learning and AI methods for modeling the progression of pregnancy. We found that deviations from a normal ‘clock’ of physical activity and sleep changes during pregnancy are strongly associated with pregnancy outcomes. When our model underestimates GA, there are 0.52 fewer preterm births than expected (*P* = 1.01e − 67, permutation test) and when our model overestimates GA, there are 1.44 times (*P* = 2.82e − 39, permutation test) more preterm births than expected. Model error is negatively correlated with interdaily stability (*P* = 0.043, Spearman’s), indicating that our model assigns a more advanced GA when an individual’s daily rhythms are less precise. Supporting this, our model attributes higher importance to sleep periods in predicting higher-than-actual GA, relative to lower-than-actual GA (*P* = 1.01e − 21, Mann-Whitney U). Combining prediction and interpretability allows us to signal when activity behaviors alter the likelihood of preterm birth and advocates for the development of clinical decision support through passive monitoring and exercise habit and sleep recommendations, which can be easily implemented in LMICs.

## Introduction

Preterm birth is the single largest cause of death in children under five^1^. While there is a plethora of research trying to build predictive models to estimate the risk of preterm birth based on various data, few effective, inexpensive, and low-risk interventions exist^2–4^. Broadly, there remains a preponderance of clinical, biological, genetic, sociodemographic, and environmental factors that are known to influence the trajectory of pregnancy^3^.

Wearable devices that directly measure physical activity have been shown to be capable of measuring stress-related variables, such as sleep quality^5–7^. Given that health statuses relevant to preterm birth can be measured from data on sleep and activity that is inexpensive to collect, albeit difficult to interpret, we hypothesized that developing predictive models from wearables data may offer a unique opportunity for low-risk and cost-effective interventions into reducing preterm birth.

While wearable devices that monitor physical activity have been used to assess sleep quality and a growing number of health-related variables, their effective integration into clinical workflows remains challenging^6,7^. One problem lies in the difficulty of analyzing wearable data, which is collected and recorded continuously, and of long-length and low dimensionality, which makes it difficult to identify the role that wearables should play in addressing at-risk behaviors^6^. Existing analytical methods for wearables-derived accelerometry data rely on black-box commercial toolboxes, bespoke pipelines for narrow applications, or non-parametric techniques with limited scope^8^. Given that measuring physical activity and sleep quality is difficult and questionnaires to associate wearables data with activity and sleep quality lack resolution over time and are subjective and inaccurate, we sought to develop a more general analytical pipeline. In particular, we sought to analyze wearables data to uncover existing relationships between stress and inflammation, and elevated risk of preterm birth.

Disruptions to one’s circadian rhythm may affect the timing of birth and negatively impact fetal and maternal health^9,10^. Few studies have explored the utility of using wearables data to extensively monitor pregnancy in humans, and even fewer in conjunction with chronodisruption. As a result, the extent to which physical activity and sleep changes during pregnancy remains largely unknown. Here, we used data from a unique cohort utilizing wearable devices and a novel state-of-the-art deep learning and inference pipeline to define, for the first time, the dynamic changes that occur to sleep and physical activity during pregnancy.

Our method, *series2signal*, differs from existing approaches in several ways. Prior state-of-the-art time-series classification models do not extensively use data augmentation techniques to improve performance^11,12^, despite its demonstrated utility in other machine learning domains^13^. In series2signal, we developed an automatic and data augmentation pipeline applied to each minibatch that is compatible with any time-series data. Additionally, we developed a new deep learning architecture for time-series classification and regression based on a model that was inspired by ResNet architecture^11,14^. To do so, we adapted bottleneck and convolutional layers to optimally capture information at time-intervals consistent with wearable device data. Finally, although prior works have applied machine learning to wearables data, we developed a novel and extensive post-hoc inferential and analysis pipeline. This pipeline automates downstream tasks to build on our deep learning model’s hidden representations and associate various model representations and outputs with maternal health outcomes and clinical measures^15^.

In this study, we applied series2signal to a cohort of pregnant study participants given wearable devices throughout their pregnancy to discover whether deviations from sleep and activity patterns are associated with PTB and other poor pregnancy outcomes. Our series2signal inference and analytical pipeline allowed us to combine prediction from wearables data with explainable AI to interpret model output as a robust signal of activity and sleep behaviors that are associated with preterm birth and other pregnancy outcomes.

series2signal demonstrates the promise of using wearables data and computation to personalize and identify the likelihood of preterm birth based on relatively inexpensive data. Our results suggest that consultation and intervention, informed by continuous monitoring, may enable cost-effective and scalable mitigations to the unacceptably high prevalence of preterm birth globally. This study serves as a platform for future randomized controlled trials to study the causation of the associations identified as well as investigations into possible implications for the immune system^2,4,16–18^.

## Results

### Data collection and cohort

**Fig. 1:**
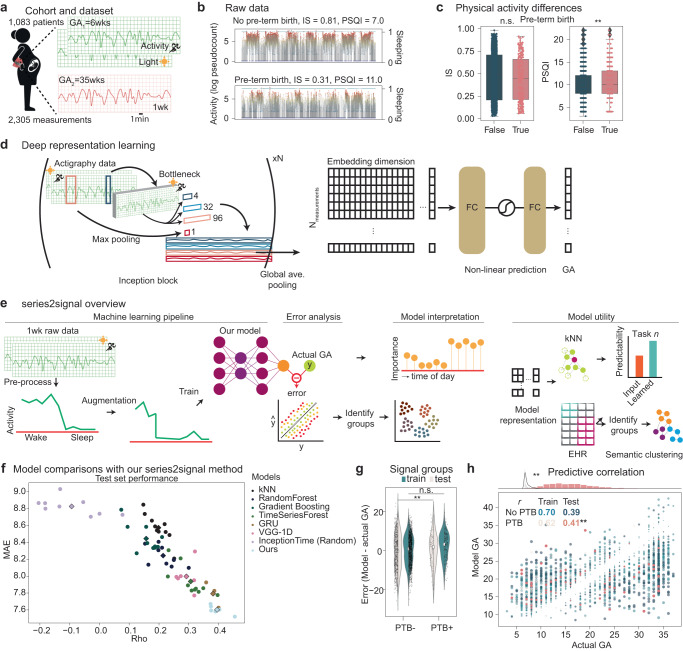
Overview of series2signal, a method to monitor pregnancy by applying deep learning to data from wearable devices. **a** *N* = 1083 patients were monitored during pregnancy. **b** Examples of pre-processed actigraphy data drawn randomly from a pregnancy that resulted in preterm birth (bottom) and a term pregnancy (top), as well as the interdaily stability (IS) and Pittsburgh Sleep Quality Index (PSQI) values for each patient. Green dots indicate binary sleep periods (right axis) per timepoint while dot color on the traces indicate the intensity of activity at a particular instance (left axis). **c** The distribution of IS and PSQI in the full cohort for preterm birth (PTB+) or term birth (PTB-). **d** series2signal model architecture showing the number of Inception blocks (in this study, *N* = 9), and 1D convolutional filters applied to the multivariate time-series input followed by global average pooling to a non-linear representation of actigraphy data that we feed through an adapted prediction block to output a prediction of GA given 1wk of actigraphy data. **e** The series2signal machine learning pipeline, with a novel data augmentation scheme to limit over-fitting, and associated modules for error analysis, model interpretation in-light of associated error groups and metadata, and model utility with respect to predictability of series2signal’s learned representations and semantic clustering for phenotyping patients based on their actigraphy data. **f** series2signal outperforms various machine learning methods and is significantly better than random, suggesting that we can monitor pregnancy from wearables data alone. Mean absolute error (MAE) of the model relative to the actual GA of measurements in the test set versus the correlation between the model’s prediction and actual GA is visualized. Diamond markers indicate the average across trials, which are represented as dots in the scatter-plot. **g** Comparison of the error of the top-1 series2signal model on this cohort showing over-fitting between the train and test sets for pre-term birth vs. the term birth group. **h** series2signal top-1 predicted GA versus actual GA for the train set (scatter-plot) with Spearman’s *ρ* correlation shown for each group split by PTB+/PTB- and train/test set (colors). Point size is scaled by the absolute error of that observation (model output - actual). Significance test encoding is for Mann-Whitney U or Spearman’s *ρ* where `**' indicates 0.001 < *P* < 0.01. Box plots show median and first and third quartiles with outliers as 1.5 times inter-quartile range (IQR).

To assess whether the combination of machine learning and wearable device data can be used to monitor the progression of pregnancy, we collated a dataset from a cohort of *N* = 1083 pregnant individuals (see Fig. 1a). Wearable actigraphy devices, which capture physical activity and motion, were given to pregnant individuals in their first trimester. Their gestational age was predominantly measured by a combination of last menstrual period (LMP) and < 14 weeks ultrasound (*N* = 537(49.6%)). From follow-up visits, we truncated actigraphy data to capture 1 week of actigraphy data post-GA indication. This resulted in *N* = 2305 data points capturing activity and sleep behaviors during pregnancy. Actigraphy data consist of counts to a piezoelectric device, measuring intensity of acceleration, integrated across 3 dimensions every minute. The actigraphy devices also collect light intensity via a photoelectric sensor, yielding a 2-dimensional time-series data set at 1-min periods across 1-week (length *L* = 10,080, and dimensionality *d* = 2). This medium-sized labeled dataset allowed us to develop a supervised machine learning pipeline to monitor pregnancy from wearable devices.

Electronic health record data was collected along with physical activity data (see Table 1). Across *N* = 2305 measurement-GA pairs, the median GA was 21.0 weeks (inter-quartile range (IQR), 11.0−30.0 weeks). Consistent with previous epidemiological studies, we found that between pregnancies that resulted in prematurity and those that came to term, BMI, history of prior PTB, and the presence of comorbidities such as hypertension and diabetes were higher for measurements associated with patients whose pregnancy resulted in PTB (*P* < 0.001, all, Kruskal-Wallis or chi-squared). The chances of C-section, neonatal complications, and death were also markedly higher amongst patients in the PTB+ group, as expected (minimum enrichment between PTB+ vs. PTB-, 45.4%, all *P* < 0.001).

**Table 1:** Cohort variables of interest stratified by whether the pregnancy measured resulted in preterm birth or not.

Variable	All	No pre-term birth	Pre-term birth	*P*_*corrected*_	Effect
	*N* = 2305; *N*_*pt*_ = 1083	*N* = 2028; *N*_*pt*_ = 930	*N* = 277; *N*_*pt*_ = 153		
Age (y)	29.00 (25.00–32.00)	29.00 (25.00–32.00)	29.00 (25.00–33.00)	1.00e+00	1.98
BMI (V1)	26.78 (22.92–33.47)	26.30 (22.72–32.91)	30.60 (24.47–36.73)	3.82e-09***	42.05
Prior PTB	296 (12.84)	200 (9.86)	96 (34.66)	3.28e-27***	−23.20 (False) 169.88 (True)
Prior FTB	1176 (51.02)	1024 (50.49)	152 (54.87)	1.00e+00	−1.03 (False) 7.55 (True)
No alcohol	2186 (94.84)	1918 (94.58)	268 (96.75)	1.00e+00	−0.28 (False) 2.02 (True)
Hypertension	229 (9.93)	159 (7.84)	70 (25.27)	1.50e-15***	−21.08 (False) 154.36 (True)
Pregestational diabetes	54 (2.34)	36 (1.78)	18 (6.50)	8.24e-03**	−24.23 (False) 177.38 (True)
Diabetes	121 (5.25)	86 (4.24)	35 (12.64)	1.83e-05***	−19.22 (False) 140.70 (True)
Heart disease	63 (2.73)	51 (2.51)	12 (4.33)	1.00e+00	−7.99 (False) 58.50 (True)
Depression	326 (14.14)	257 (12.67)	69 (24.91)	1.30e-04***	−10.40 (False) 76.13 (True)
Other disease	573 (24.86)	436 (21.50)	137 (49.46)	1.13e-19***	−13.52 (False) 98.96 (True)
Gestational diabetes	119 (5.16)	97 (4.78)	22 (7.94)	1.00e+00	−7.35 (False) 53.84 (True)
Preeclampsia	264 (11.45)	163 (8.04)	101 (36.46)	2.96e-40***	−29.82 (False) 218.35 (True)
Corticosteroids	270 (11.71)	72 (3.55)	198 (71.48)	8.66e-234***	−69.69 (False) 510.23 (True)
Gynecological infection	963 (41.78)	833 (41.07)	130 (46.93)	1.00e+00	−1.68 (False) 12.33 (True)
Epidural	1824 (79.13)	1641 (80.92)	183 (66.06)	3.39e-05***	2.26 (False) -16.51 (True)
C section	727 (31.54)	600 (29.59)	127 (45.85)	8.95e-03**	−6.20 (False) 45.37 (True)
Infant weight (kg)	3200.00 (2830.00–3590.00)	3280.00 (2977.50–3630.00)	2340.00 (1880.00–2750.00)	9.92e-93***	424.71
Death of baby	42 (1.82)	7 (0.35)	35 (12.64)	6.49e-42***	−81.06 (False) 593.44 (True)
Neonatal complication	518 (22.47)	351 (17.31)	167 (60.29)	2.42e-54***	−22.98 (False) 168.27 (True)
ER visit w/in 4 weeks	193 (8.37)	165 (8.14)	28 (10.11)	1.00e+00	−2.83 (False) 20.72 (True)
V1 sleep quality (= very good)	281 (12.19)	266 (13.12)	15 (5.42)	1.00e+00	7.59 (False) -55.58 (True)
IS	0.48 (0.20–0.70)	0.49 (0.20–0.71)	0.44 (0.21–0.66)	1.00e+00	2.88
IV	0.43 (0.34–0.56)	0.43 (0.34–0.56)	0.45 (0.34–0.57)	1.00e+00	0.12
RA	0.78 (0.70–0.85)	0.78 (0.70–0.85)	0.76 (0.64–0.86)	1.00e+00	4.86
Minutes rest (in 1 week)	6109.00 (5178.00–7993.00)	6113.50 (5183.50–7999.25)	6085.00 (5125.00–7921.00)	1.00e+00	0.19
< activity >_wake_	5.26 (5.12–5.41)	5.26 (5.12–5.41)	5.27 (5.13–5.43)	1.00e+00	0.93
< activity >_sleep_	0.48 (0.21–0.65)	0.48 (0.20–0.65)	0.51 (0.23–0.64)	1.00e+00	0.07
< activity >_weekend_	2.31 (0.71–2.88)	2.31 (0.68–2.89)	2.29 (1.01–2.86)	1.00e+00	0.04
< activity >_weekday_	2.48 (1.41–2.98)	2.48 (1.41–2.98)	2.49 (1.41–3.03)	1.00e+00	0.41
PSQI	10.00 (8.00–12.00)	10.00 (8.00–12.00)	10.00 (8.00–13.00)	2.83e-01	7.38
KPAS	9.35 (7.96–10.55)	9.35 (7.95–10.58)	9.36 (8.11–10.36)	1.00e+00	0.16
EpworthSS	7.00 (4.00–11.00)	7.00 (4.00–11.00)	8.00 (4.00–12.00)	1.00e+00	3.81
Edinburgh	3.00 (0.00–6.00)	3.00 (0.00–6.00)	4.00 (1.00–9.00)	1.50e-03**	17.13

P values were corrected for multiple comparisons (Bonferonni correction with *N* = 87) and represent Kruskal-Wallis tests for continuous variables or chi-squared tests for categorical variables between preterm birth positive and negative groups.

*PSQI* Pittsburgh Sleep Quality Index, *KPAS* Kaiser-Permanente Activity Scale, *EpworthSS* Epworth Sleep Scale for daytime sleepiness. See section below for description of activity metrics. For continuous variables, the effect is the Kruskal-Wallis test statistic and for categorical variables, the effect is the χ^2^ contingency table test observed vs. expected (OE) ratio minus 1 for the PTB+ (True) and PTB- (False) group. Significance key: *P* > 0.05: ‘n.s.’, *P ≤ 0.05, **P ≤ 0.01, ***P ≤ 0.001.

### Self-reported data and standard quantification of actigraphy data do not identify signal associated with GA or PTB

Standard physical activity monitoring analyses rely on comparisons of non-parametric measures across groups^8^. For example, circadian rhythmicity can be assessed in PTB+ vs. PTB- pregnancies via day-to-day variance (IS=interdaily stability), activity fragmentation (IV=intradaily variability), and the relative difference between the mean activity during the 10 most active hours and the 5 least active hours (RA=relative amplitude). Standard actigraphy analytical methods, which use non-parametric activity-related summary metrics, revealed no difference between preterm and non-preterm births (PTB+ vs. PTB- groups, minimum *P*_*corrected*_ = 1.18 for RA, Kruskal-Wallis).

Standard activity metrics can be correlated with each other, e.g., activity and sleep (Spearman’s *ρ* = 0.41(*P* < 0.001) for association between Kaiser Permanente Activity Scale (KPAS) and PSQI in total cohort; and across groups, *ρ*_*PTB*+_ = 0.36(*P* < 0.001) and *ρ*_*PTB*−_ = 0.41(*P* < 0.001); see Supplementary Fig. 2A). In addition to subtle differences between pre-term birth groups in this cohort, non-parametric sleep and activity metrics mostly do not correlate with progression of pregnancy (see Supplementary Fig. 2C); however, IS and IV are weakly correlated with GA (max *ρ* = 0.19(*P* < 0.001), IS) but do not significantly differ between pre-term birth groups (Spearman’s *ρ*_*PTB*+_ = 0.15(*P* = 0.01) and *ρ*_*PTB*−_ = 0.20(*P* < 0.001), IS). This suggests that non-parametric activity monitoring metrics cannot be robustly used to assess prematurity risk on an individual patient-level. Instead, the combination of non-parametric physical activity monitoring analyses and wearables data during pregnancy supports that pregnancy disrupts circadian rhythmicity, as has been reported elsewhere^9,10^. From visual comparison of raw actigraphy data traces, the perplexity of analysis strategy for this data is more pronounced; furthermore, group comparisons reveal no significant differences between measurements associated with individuals whose birth results in pre-term or term (Fig. 1b, c). While survey data indicates there may be some differences in PTB+/PTB- in terms of sleep quality and depression scales, these effect sizes are small (maximum median difference of 1 point in the Edinburgh depression scale, higher for PTB+), reiterating that the complexity of the data requires additional analytical techniques to maximize the utility of collecting physical activity monitoring data during pregnancy.

### series2signal identifies sleep and activity patterns associated with the progression of pregnancy with deep learning

To support the integration of information distilled from wearables into pregnancy monitoring workflows, we developed a new machine learning and explainable AI pipeline, dubbed *series2signal*, which ingests periods of wearables data from actigraphy devices and builds a model of dynamic physical activity and sleep changes during pregnancy. We further demonstrate that deviations from this “normal” model signals disruptions to physical activity and sleep, relative to healthy, and are associated with adverse outcomes.

Our series2signal method has four primary components (see Fig. 1e): (1) a machine learning pipeline, which includes data cleaning and pre-processing, a new model for multivariate time-series regression using a convectional-based deep learning architecture (Fig. 1d), and a new time-series data augmentation scheme; (2) a trained model error analysis and automated tabular correlation network function that identifies sub-groups within error modes and clinical (or metadata) groups; (3) a feature-based attribution module to interpret the trained model and address why, on the basis of time-of-day and clinical group, the model is making its predictions; (4) a lightweight addendum to the model that can be used for predicting sub-group membership or other ancillary tasks based on the trained models’ representations and metadata, and phenotyping (relative to the trained cohort) based on the models’ learned representation (see Fig. 1e and methods).

### series2signal outperforms standard machine learning approaches

Our machine learning pipeline achieves the best average and maximal performance on predicting GA from actigraphy data relative to other deep learning and machine learning approaches (Fig. 1f, Table 2). In particular, on a held-out test set, series2signal achieves a minimum of average absolute error of 7.52 weeks and, across the test set, the model’s GA output correlates with the actual GA (Spearman’s *ρ* = 0.45(*P* < 0.001)). Our model achieves the absolute-lowest error on the test set of all comparison models tried. In head-to-head comparisons with repeated trials (in which were robustness tests were performed by assigning patients to different training, testing, and validation splits per trial), series2signal consistently out-performs all other models and is significantly better than all other ML/DL models in head-to-head comparisons (Table 2). In particular, relative to a random model trained with the series2signal architecture on randomized targets, ours achieves the largest top-1 difference, with 18.1% lower error. On average, the non-deep learning TimeSeriesForest model generalizes with a sub-2 month error on GA prediction; however, the series2signal model still outperforms this and other ML methods with only ~ 2000 actigraphy measurement - GA pairs (max *P* = 0.0001, Mann-Whitney U). Furthermore, the output of the TimeSeriesForest is significantly less correlated with the actual GA than the series2signal output, on average across trials (*ρ*_*Ours*_ = 0.40 vs. *ρ*_*TimeSeriesForest*_ = 0.31). This suggests that series2signal is, to-date, the best method to monitor pregnancy using just 1 week of wearables data.

**Table 2:** Head-to-head comparison of our model for predicting gestational age with other top ML methods and DL architectures for time-series.

Model	MAE	Top-1 diff (MAE)	P (MAE)	*ρ*	Top-1 diff (*ρ*)	P (*ρ*)
kNN	8.57 (0.16)	−0.18 (v.InceptionTime (Random))	1.16e-03** (v.InceptionTime (Random))	0.20 (0.03)	−0.20 (v.Ours)	1.67e-08*** (v.Ours)
RandomForest	8.24 (0.11)	−0.63 (v.InceptionTime (Random))	1.90e-09*** (v.InceptionTime (Random))	0.21 (0.04)	−0.15 (v.Ours)	3.57e-07*** (v.Ours)
Gradient Boosting	8.45 (0.15)	−0.24 (v.InceptionTime (Random))	1.15e-05*** (v.InceptionTime (Random))	0.16 (0.04)	−0.24 (v.Ours)	2.13e-08*** (v.Ours)
TimeSeriesForest	7.94 (0.17)	−0.72 (v.InceptionTime (Random))	2.79e-10*** (v.InceptionTime (Random))	0.31 (0.05)	−0.06 (v.Ours)	2.00e-03** (v.Ours)
GRU	7.79 (0.12)	−1.11 (v.InceptionTime (Random))	8.17e-08*** (v.InceptionTime (Random))	0.38 (0.02)	−0.05 (v.Ours)	5.28e-01 (v.Ours)
VGG-1D	8.00 (0.15)	−0.74 (v.InceptionTime (Random))	2.00e-08*** (v.InceptionTime (Random))	0.29 (0.03)	−0.13 (v.Ours)	1.70e-04*** (v.Ours)
InceptionTime (Random)	8.83 (0.09)	0.18 (v.kNN)	1.16e-03** (v.kNN)	−0.09 (0.09)	−0.38 (v.Ours)	2.47e-08*** (v.Ours)
Ours	7.60 (0.05)	−1.36 (v.InceptionTime (Random))	7.39e-13*** (v.InceptionTime (Random))	0.40 (0.03)	0.05 (v.GRU)	5.28e-01 (v.GRU)

*MAE*, Mean absolute error, *Rho*, Spearman’s correlation coefficient. *n* = 10 trials were conducted for non-deep learning methods, and *n* = 6 trials for deep learning methods except for GRU, which was only run with *n* = 3 trials. For each trial, new dataset splits were sampled to combine robustness and stochasticity tests per model. The *P*-value per metric and comparison is assigned by a t-test. Significance key: *P* > 0.05: ‘n.s.’, *P ≤ 0.05, **P ≤ 0.01, ***P ≤ 0.001.

With small training size (*N*_*measurements*,*train*_ = 1411 vs. *N*_*measurements,test*_ = 691), we expected to suffer from over-fitting. To address this, we developed a new procedure for data augmentation to mitigate this problem. We selected a number of augmenting filters and applied them all or randomly selected one per mini-batch or epoch, optimizing the procedure to minimize generalization error. Including this data augmentation in our training implementation, we mitigated over-fitting, obtaining similar performance on the train and test set (see Methods, compare train/test error in Fig. 1g, h). However, stratifying the model output on whether the pregnancy resulted in full-term or pre-term birth reveals that the model’s error (model minus actual GA) is significantly higher in the pre-term birth group compared to the non-preterm birth group in both training and validation splits (mean difference in error, 2.31 weeks, *P* = 0.005; Fig. 1h). In particular, stratifying on whether the measurement is associated with a pregnancy that did or did not result in a preterm birth, we find that the top-1 model output on the train set is correlated with the actual GA in both groups, which is maintained in the test set (*ρ*_*PTB*−_ = 0.39 vs. *ρ*_*PTB*+_ = 0.41, max *P* = 2.1e − 4, test PTB+) Fig. 1g). Thus, for the groups of interest, the model performs nearly as well, suggesting that the top-1 model can address differences between PTB groups.

### Deviations from the activity and physical clock of pregnancy is associated with adverse outcomes

**Fig. 2:**
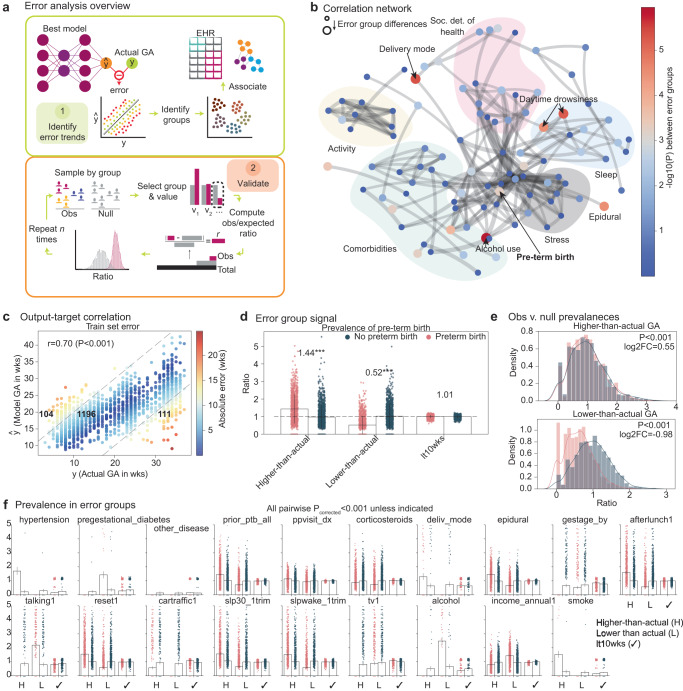
series2signal model error is associated with increased likelihood of adverse pregnancy outcomes. **a** Overview of series2signal model that takes the top-1 series2signal model to identify trends in the model’s idiosyncrasies and determine the extent of association between error groups and metadata groups. The validation of identified error trends using a permutation testing scheme on the test set. **b** Correlation network where the node-size shows error group differences, determined by the magnitude $-{\log }_{10}{P}_{{{{\rm{corrected}}}}}$, where edges are cut off at *ρ* = 0.6 and thickness is proportional to the strength of association. Node categories are manually drawn and detailed in the supplement. **c** Top-1 series2signal model predictions for GA versus actual GA on the train set, where bold numbers show the number of measurements in each error group and Spearman’s *ρ* shows the overall correlation between predicted and actual GA. **d** The observed versus expected ratio divided by the expected ratio for each error group in PTB+ vs. PTB- groups where the number above the preterm birth group shows the enrichment or decrease in prevalence relative to expected for the train set. **e**, **f** Color indicates PTB+/− group membership in accordance with (**d**). **e** Permutation tests of ratios between PTB+/PTB- negative groups in the higher-than-actual (top) and lower-than-actual (bottom) error group, with the ${\log }_{2}$ fold-change between means of each distribution shown with a Mann-Whitney-U *P*-value in the legend. **f** comparison of permutation tests for other metadata groups. All P-values for comparison of the observed metrics (pink), relative to the permutation test null distribution (blue) are significant (*P* < 0.001) unless otherwise indicated. Bar plots show mean and error bars represent the standard deviation across the distribution of computational experiments for the indicated metric.

series2signal includes a new pipeline to analyze the best performing model (top-1 model), comparing its predicted GA output to the actual output and analyzing the association of that error with clinical variables. The error analysis module consists of two parts: (1) first, we identify error trends with an automated process that leverages a correlation network and the metadata or clinical data associated with each patient; (2) then, we assess the significance of these groupings and error group differences using permutation testing and determine which differences generalize to the test set (Fig. 2a). By comparing the model output to the actual GA, we identify at least three error modes: measurements where the predicted GA is higher than the actual GA (higher-than-actual GA group), measurements where the predicted GA is lower than the actual GA (lower-than-actual GA group), and a small error group ( < 10*weeks*, or *lt*10*wks*, where the threshold determined by balancing the number of samples in the higher- and lower-than-actual error modes; Fig. 2c). These error groups are also present in similar proportions in the held-out test set, suggesting that these error groups can be identified with series2signal (Supplementary Fig. 3A). With these error groups, we can automatically associate each error group with metadata or clinical data available per patient via a custom tabular association function that accounts for mixed categorical and continuous associations (see Methods). Once we have these error groups, in the training and test set we sample from each error mode and shuffle the group labels to create an observed and null set. Then, via permutation testing, we can compare whether the observed versus expected ratio (where the expected ratio uniformly mixes a particular label across groups) is significantly different, allowing us to validate whether the observed differences amongst error groups are robust.

We leverage *m* = 124 metadata variables, spanning patient-level data on stress and depression, surveys and scores on sleep and activity, social determinants of health, and various clinical outcomes, comorbidities, and procedural details, comparing and contrasting the differences within each variable across error groups. This yields a correlation network where the node size is given by differences between groups (by the negative logarithm of the p-value derived from statistical hypothesis testing between groups, namely, ANOVA or Fisher’s exact test for continuous or categorical data, respectively; Supplementary Fig. 3B, C). This correlation network, unsupervised, clusters variables tracking similar quantities, e.g., activity-related variables, together, highlighting its ability to reflect health-relationships consistent with common sense understanding. The strongest and most numerous interactions occur between sleep, comorbidities, and social determinants of health, while activity and stress are more distally related, albeit to one another. One of the largest differences across groups occurs in the prevalence of preterm birth. Other important clinical variables differ across error groups, including whether the patient used alcohol during pregnancy, whether the patient asked for an epidural, a few sleep quality metrics, the delivery mode of the birth, and the presence of hypertension during pregnancy.

Differing across error groups, however, does not necessarily portend significance. To ensure that these clinical variables differing across error groups also can be modeled, reliably, from actigraphy data, we compare both the difference in prevalence or value for each metadata variable against its difference across error group (Supplementary Fig. 3B). This shows that while there are differences in the amount with which epidurals are received across the error groups, it is hard to model this using actigraphy data, which may suggest that the model error analysis may not as robustly identify these modes across error groups, given significant differences in the cohort but poor accuracy from input data for the model. In contrast, hypertension and sleep metrics and, in particular, the outcome of preterm birth, all differ across error groups and have high predictability. This suggests that preterm birth differing across error groups is a detectable signal using series2signal. Indeed, the largest magnitude of difference for pregnancy outcome across error groups is infant length, which is itself known to be correlated with preterm birth (Supplementary Fig. 5A). In addition, several sleep-quality-related variables differ across error groups, which suggests that disruption to sleep-quality is associated with model error, which is in turn associated with differences in pregnancy outcome. Activity-related metadata variables are highly predictable from actigraphy data (Supplementary Fig. 3B), while other categories of metadata are less predictable, reinforcing the utility in setting the primary task of series2signal to modeling of GA. The high-predictability of social determinants of health and differences across error groups, however, may suggest an association between sleep and activity and model output.

We further explore and validate the assertion, based on initial results, that the model’s output, “actigraphy-GA” itself can be compared with the actual GA to indicate deviations to sleep and activity and indicate a heightened risk for preterm birth and adverse outcome *and* a decreased risk of preterm birth and adverse outcome when the model thinks the pregnancy is not as far along as it is (namely, when the “actigraphy-GA” is lower-than-actual GA). Based on the proportion of samples in each group and the prevalence of a particular value, *e.g*., whether a pregnancy resulted in preterm birth or not, we can use a *χ*^2^ metric to compare the observed to expected ratio. For example, if there were *n* = 100 preterm births and the proportion in each error group was 80/10/10%, then we would expect to observe *n* = 80, *n* = 10, and *n* = 10 in the respective groups, each with an observed/expected ratio = 1. Instead, we observe that when the model outputs an “actigraphy-GA” that is higher-than-actual (the model thinks the pregnancy is further along than it actually is), there is a 44% increased prevalence of preterm birth in that error group (Fig. 2d). In contrast, the prevalence of preterm birth is reduced by more than a factor of 2 (0.52*X* expected) when the model thinks the pregnancy is less far along than it actually is (lower-than-actual error group). In the low-error mode, the prevalence of preterm birth does not differ significantly from expected by chance. To assess the significance of this result, we performed permutation testing, finding that after repeated sampling from the held-out test set, the model’s output can consistently be used to indicate higher prevalence of preterm birth for higher-than-actual GA estimations (prevalence of preterm births is higher by a factor of 1.5 than expected, *P* < 0.001). On the test set, the model’s output consistently can be grouped into a lower-than-actual GA group where the prevalence of preterm birth is less-than-expected by a factor of 2.0 (*P* < 0.001, permutation test) (Fig. 2e). Thus, while the model’s error may have a high-magnitude in some cases, the placement of a week-long measurement into a high-error group can indicate deviation to sleep and activity that portend heightened (or reduced) risk of preterm birth.

We perform similar analyses for metadata variables that significantly differed across error groups (Fig. 2f). However, given the small size of the dataset and error groups ($\sim {{{{O}}}}(2)$) and the rarity of some conditions, e.g., pregestational diabetes, there are not always examples of the metadata variable value being observed across all error modes, i.e., the power is low. Nonetheless, some trends are noticeable, such as the prevalence of a postpartum diagnosis (pre-term birth, neonatal complication) being significantly higher in the higher-than-actual GA error group and significantly lower in the lower-than-actual GA error group (*P* < 0.001, both, permutation test). Generally, several sleep-related variables, pregnancy outcome variables, and social determinants of health significantly differ across error groups, suggesting that series2signal may pick up a number of broadly and clinically relevant signals from just 1wk of wearable device or physical activity and sleep monitoring.

In our cohort, some individuals may have had iatrogenic preterm births, where delivery was scheduled before GA=37 weeks due to maternal and/or fetal causes. Induction was indicated for various reasons in our cohort, including pre-eclampsia, individual election, intrauterine growth restriction, fetal demise, gestational diabetes, and others^19^. To assess the sensitivity of our primary results to spontaneous vs. iatrogenic births, we split our dataset into two groups: one in which induction is indicated for any reason and another group, representing spontaneous births, where no induction is indicated for the delivery of the pregnancy (*N*_no induction_ = 623 and *N*_induction indicated_ = 460; see Supplementary Fig. 4A). In both subgroups, series2signal model output remains correlated with actual GA (*ρ* = 0.61 and *ρ* = 0.57 for no induction and any induction indicated, *P* < 0.001 both, see Supplementary Fig. 4B, E). Average series2signal model error is also higher in PTB+ for both groups (0.3 vs. 2.5 weeks, *P* < 0.001 and 0.1 vs 2.6 weeks, *P* = 0.001, for spontaneous and induction groups respectively in the training set, Mann-Whitney U; see Supplementary Fig. 4D, G), consistent with our findings for the full cohort. Lastly, trends in error groups remain consistent with results on the full cohort (OR for enrichment of PTB+ in higher-than-actual error 1.13 vs. 1.81 for spontaneous vs. any induction births, *P* = 9.7e − 6 vs. *P* = 6.7e − 55 by permutation tests, respectively, and OR for enrichment of PTB+ in lower-than-actual error group is 0.45 vs. 0.87 in spontaneous vs. any induction births, *P* = 2.3e − 137 vs. *P* = 5.0e − 6, respectively; see Supplementary Fig. 4C, F). These results suggest that series2signal can be used to indicate risk of preterm spontaneous or iatrogenic birth based on physical activity, sleep, and light exposure patterns and behavior.

### series2signal relies on deviations from general sleep and activity patterns in predicting higher- or lower-than-actual GA

**Fig. 3:**
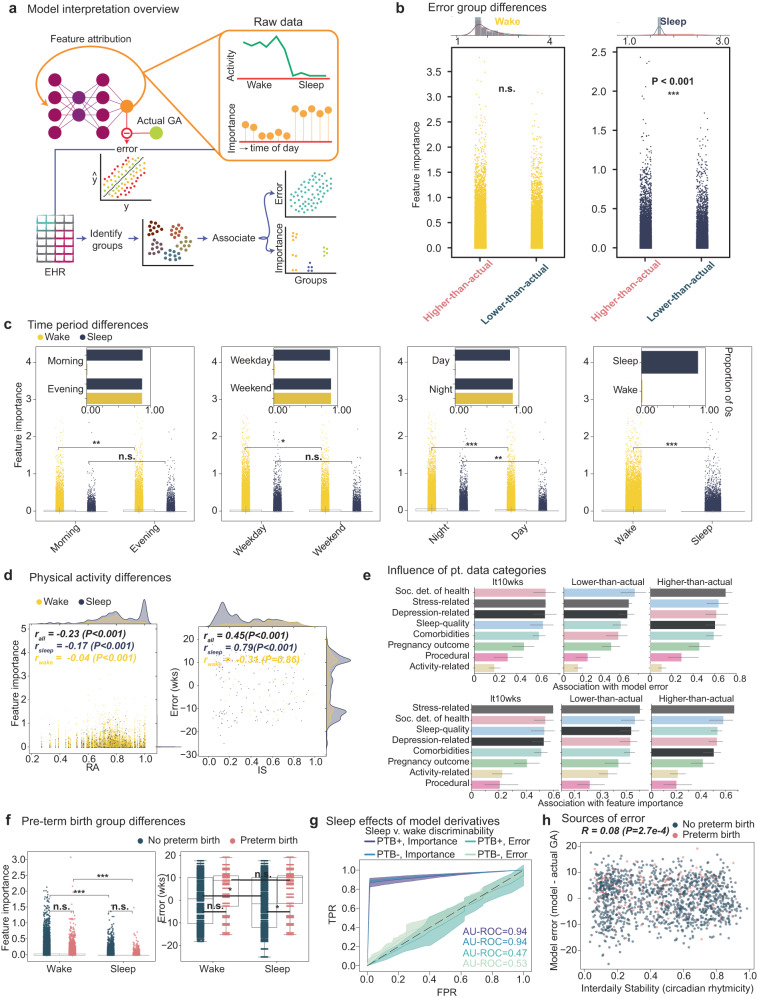
Model interpretation reveals that series2signal relies on deviations to sleep and activity in predicting higher- or lower-than actual GA error groups. **a** Use of gradient based feature attribution method to calculate feature importance per input time from the input actigraphy data using the top-1 series2signal model and its downstream association with error groups and metadata groups to search for associations between clinically relevant variables and model error and feature weighting. **b** Comparison of feature attribution scores during wake (yellow, left) and sleep (blue, right) periods between the higher- and lower-than-actual error groups. Top distribution shows the top *n* = 200 feature importance scores. **c** Comparison of feature importance scores by time-of-day or week. Inset shows the proportion of 0s (within tolerance = 1e − 8) of feature importance during the indicated period. **d** Spearman’s *ρ* correlation of feature importance or error against relative amplitude (RA) or interdaily stability (IS). **e** The average association score per metadata variable category against top-1 series2signal model error and feature importance (rows) per error group (columns). Color bar annotation is denoted in the first column and carries over, representing variable category. **f** Comparison of feature importance across PTB+ vs. PTB- groups across feature importance and model error. **g** AU-ROC curves showing true positive rate (TPR) against false positive rate (FPR) for a logistic regression classifier discriminating between sleep and wake using the continuous feature importance scores and error from PTB+/PTB- groups. Lineplot intervals show 95% confidence intervals. **h** Spearman’s *ρ* correlation between model error and IS for PTB+ vs. PTB- groups. Significance key: *P* > 0.05: `n.s.', *P* ≤ 0.05: `*', *P* ≤ 0.01: `**', *P* ≤ 0.001: `***'. Continuous variables were compared with Mann-Whitnuey U test or Spearman’s *ρ*. Barplot error bars represent standard deviation. Box plots show median and first and third quartiles with outliers as 1.5 times IQR.

Why the model error is a useful pregnancy monitoring indicator was an open question and, to ensure trustworthiness and robustness of the series2signal method, as well as the ability of future users to modify the model without losing the ability to interpret and validate its utility, we include a model interpretability module in the series2signal method. We rely on a gradient-based feature attribution method, which allows us to query each actigraphy data sample and obtain local explanations, i.e., per actigraphy data sample, which individual measurements are important in influencing the model’s predictions (Fig. 3a). With these feature attributions, we can then globally frame post-hoc model inference for interpretability across the cohort and group analyses into sub-groups identified from metadata or other means. Broadly this allows us to interrogate why the model makes its predictions and why the predictions that signal heightened (or decreased) risk of preterm birth are made, which may facilitate the development of correcting interventions. Briefly, we developed an automated method that associates the model’s error or feature attribution score (feature importance, absolute value of integrated gradient method) with clinical variables per feature and per sample group. Groups can be flexibly defined, and in our application, we include error groups, feature groups stratified by time-of-day, e.g., higher-than-actual and morning vs. evening times, respectively, and clinical groups, e.g., whether a particular measurement was associated with a preterm or full-term birth. Then, we can rely on statistical hypothesis testing to determine whether the error or feature importance differs significantly between these groups.

Comparing measurements’ feature importance in the higher-than-actual error group versus lower-than-actual error group reveals no differences during wake periods in feature attribution scores between error groups (Fig. 3b). However, during sleep periods, the feature importance scores for the higher-than-actual group are significantly higher than the feature importance scores for the lower-than-actual group (*P* < 0.001, Mann-Whitney U). This suggests that the higher-than-actual error group relies on deviations to sleep patterns to make its predictions more so than the low and lower-than-actual GA error groups, which could be interpreted as the model paying closer attention to sleep patterns in mis-predicting the progression of the pregnancy and that sleep disruption causes the model to “think” the pregnancy is further along than it actually is. When we stratify by time-of-day rather than error group, however, the model’s behavior has a less clear interpretation. For example, during wake periods, the model relies more heavily on measurements in the evening than the morning to make its GA prediction (Fig. 3c); however, for sleepy mornings or evening naps, the model does not significantly attribute influence in its prediction. Broadly, looking at the proportion of non-zero feature importance scores, the activity during the wake periods in the mornings, weekdays, and daytime broadly influence the model’s output. Indeed, for the held-out test set, activity during wake periods are more heavily attended to by the model in making its predictions, even though this trend breaks between error groups. Indeed, feature importance scores are associated with activity metrics, such as RA (Fig. 3d) but the correlation is negligible during wake periods and small during sleep periods (Spearman’s *ρ*_*sleep*_ = − 0.17 vs. *ρ*_*wake*_ = −0.04), which may suggest that large deviations to sleep periods are particularly important in the model’s predictions. The error (model minus actual GA) is significantly correlated with daily rhythmicity (IS, *ρ*_*all*_ = 0.45, *P* < 0.001) during sleep periods but not during wake periods (*ρ*_*wake*_ = − 0.31, *P* = 0.86), which may suggest that individuals with higher day-day activity variability and sleep disruption have higher error.

Broadly, we investigated the association of feature importance across all metadata variables (Supplementary Fig. 6A–D) as well as the association of error with metadata variables (Supplementary Fig. 7A–E) to explain why series2signal can be useful for pregnancy monitoring. We relied on a new, custom function for association with mixed categorical and continuous tabular data (see Methods) and scaled the association to a number between 0 and 1. This analysis revealed that surprisingly, activity-related variables are more correlated with feature importance in sleep periods, relative to wake periods, while other metadata variables are similarly associated with feature importance regardless of sleep or wake periods (Supplementary Fig. 6A). In addition, the magnitude of the model’s error is most differentially correlated with activity-related variables between sleep and wake periods (Supplementary Fig. 7A). Comparing across error groups, stress-related variables are broadly the most highly associated with feature importance and model error (Supplementary Fig. 7E, F) while the association with activity-related variables is decoupled, albeit differential across error groups. Averaging the associations across metadata variable categories highlights that sleep-quality is most associated with model error in the lower-than-actual GA error group, while social determinants of health are particularly more strongly associated with model error in the higher-than-actual GA group (Fig. 3e). Comorbidities and feature importance are also uniquely more associated in the higher-than-actual error group, which may suggest that when the model thinks the pregnancy is further along than it really is, the individual is sicker, as detectable from series2signal and wearable device data. Broadly, these results suggest that deviations from sleep and activity vary significantly across series2signal error modes and can thus be used as indicators for other clinically relevant variables, such as the presence of comorbidities.

We lastly sought to distinguish the model’s error associations and feature attributions across prematurity groups (Fig. 3f–h). In the PTB- group, the model’s output is more highly dependent on sleep than wake periods (mean difference, 4%, *P* < 0.001, Mann-Whitney U), a trend reflected in the PTB+ group as well (mean difference, 4%, *P* < 0.001). The error (model’s “actigraphy-GA” minus actual GA) is significantly higher in the pre-term birth group during sleep and wake periods (mean difference, sleep minus wake periods, 24%, *P* = 0.03) but between sleep and wake in the PTB- group, there is no difference in the error (*P* = 0.36, Fig. 3f). Digging into this more deeply, in both the PTB+ and PTB- group, sampling feature importances from this periods and predicting whether the feature importance was drawn from sleep or wake periods reveals that importance can be used to discriminate between sleep and wake periods (AU-ROC = 0.94, both) whereas the error is a much poorer discriminator of sleep vs. wake (max balanced AU-ROC = 0.53, PTB- group; Fig. 3g). This suggests that the error in sleep periods is significantly higher in the pre-term birth case and not confounded, as the importance is, by sleep and wake. We also show that model error (model-actual GA) is negatively correlated with interdaily stability (*ρ* = 0.08, *P* = 2.7e − 4, Fig. 3h), which indicates that our model assigns a more advanced GA for individuals with decreased precision of daily sleep-wake rhythms, although this effect is subtle. In broader support of this, our model attributes higher importance to sleep periods in predicting higher-than-actual GA, relative to lower-than-actual GA (*P* < 0.001, Mann-Whitney U Fig. 3b). Collectively, this suggests that the model attends particularly closely to activity patterns in sleep when over- and under- estimating the prematurity or stage of the pregnancy.

### series2signal embeddings are useful for predicting ancillary metadata tasks and phenotyping patients based on time-series clustering

**Fig. 4:**
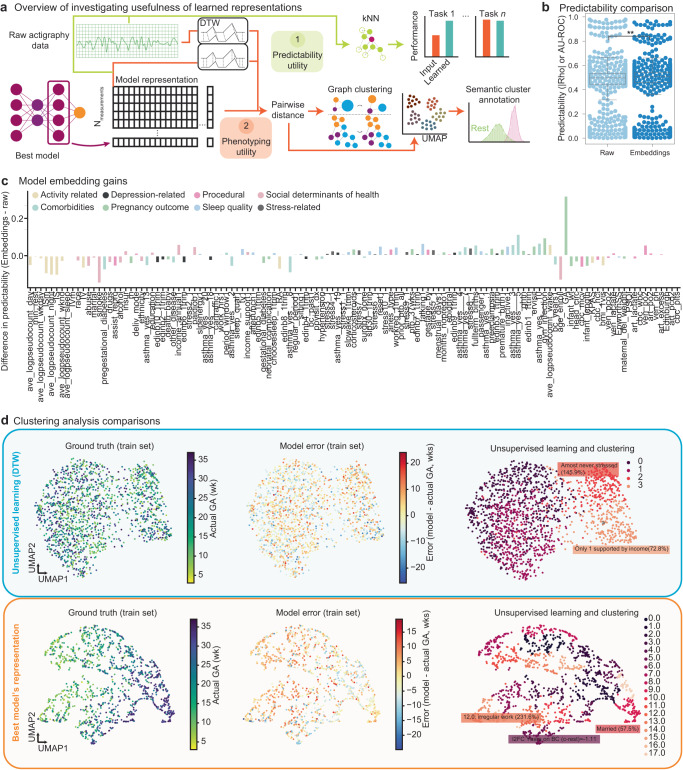
series2signal module to investigate model utility demonstrates that series2signal embeddings are useful for predicting ancillary metadata tasks and for phenotyping patients. **a** Overview of model utility module that compares an unsupervised learning approach harnessing dynamic time warping (DTW) to measure distances between pairs of time-series with supervised learning and graph clustering approach based on representations of input actigraphy data that our model learns. **b** Predictability, defined as AU-ROC for classification tasks and the absolute value of Spearman’s *ρ* for regression tasks, of metadata tasks using input, pre-processed actigraphy data (Raw) or learned model embeddings (Embeds). Box plots show median and first and third quartiles with outliers as 1.5 times IQR. **c** The difference in predictability between models built using learned model representations (Embeddings) minus those built with input actigraphy. Color shows category of metadata target. **d** Dimensionality reduction of actigraphy data using DTW (top row) versus model embeddings (bottom row) with individual measurement-GA pairs in the train set colored by actual GA, error, and graph-based clustering with semantic identification of cluster enrichment across metadata variables (columns). Far right numbers indicate cluster ID.

Deep learning models learn non-linear representations of the input data that can be then used for predicting the primary task. We tested whether the embeddings that we learned in using actigraphy data to monitor the progression of pregnancy would be useful in other tasks, namely predicting other metadata variables given wearables device data (Fig. 4a). The ability to extract embeddings depends on the construction of the network, in part. We adapt a state-of-the-art deep learning for time-series architecture in a way that allows us to add linear layers for regression tasks, in addition to providing non-linear embeddings that summarize the entire week of actigraphy data into a vector (Methods, Fig. 1a). Once we have an optimally trained model (top-1), series2signal can then output time-series or actigraphy embeddings via an input query, where the input query is the pre-processed actigraphy data from any given patient. To benchmark and compare the utility of using model embeddings we took two primary approaches: we developed an automated unsupervised clustering pipeline for comparing phenotyping based on the pre-processed actigraphy data that utilizes dynamic time warping as a distance metric for the time-series actigraphy data, and then we use a high-dimensional distance metric (Manhattan distance) to compute pairwise distances for samples’ learned embeddings, where “learned embeddings” refer to the top-1 model’s non-linear representation of the long time-series in a 128-dimensional vector. Using these pairwise distances, we can construct a graph via affine transformation and identify communities; we can visualize the high-dimensional embedding using standard dimensionality reduction algorithms, such as UMAP, and then finally, parse through the metadata to search for enrichment of meaningful value instantiations in each cluster. To assess the predictability of series2signal’s model on other clinically relevant tasks, despite training the model only to monitor the progression of pregnancy, we compare the same lightweight machine learning algorithm (kNN classification or regression) on various tasks using the top-1 model’s learned representation of the actigrahpy time-series versus the pre-processed actigraphy data.

We found that series2signal’s top-1 model, on the MOD cohort, learned representations that yield higher-on-average performance with a lightweight machine learning model than actigraphy data (Fig. 4b, *P* = 0.01, Mann-Whitney U). This suggests that series2signal can be used to compress actigraphy data (from ${{\mathbb{R}}}^{1\times 10,080\times 2}$ dimensional input per measurement to ${{\mathbb{R}}}^{1\times 128}$, a reduction of 160 × ) and used for various clinically relevant tasks that speed up complete re-training by ~ 10h per task, while also providing signal on the progression of pregnancy. While the average boost in predictability (absolute value of Spearman’s correlation for regression or AU-ROC for classification) using series2signal’s model embeddings is significant, the effect size is small (based on series2signal embeddings, Predictability = 0.46(0.2) vs. Predictability = 0.45(0.2) based on raw actigraphy data). There are also differences in which tasks, specifically are more or less predictable with the model’s learned representation of the input wearable device data (Fig. 4c, Supplementary Fig. 8B). In particular, series2signal’s representations excel at predicting social determinants of health, sleep-quality, comorbidities and stress-related metadata targets; however, the raw actigraphy data can be more easily used to discriminate activity-related metadata labels per measurement. This may indicate that the series2signal approach learns to reduce the impact of activity-related differences between the population which a less-useful model over-exploits, while our model learns more subtle patterns hidden in the wearable device data that are more closely related to important clinical variables, including indicators of sleep and stress that can signal heightened risk of prematurity.

Given that the model embeddings are predictable, we also wondered whether series2signal can produce quick visualizations for complex wearables device data that would give a global positioning of a patient at any stage in pregnancy, relative to the MOD cohort, where the localization is semantic or clinically useful. To benchmark series2signal’s top-1 model, we also developed an unsupervised learning approach, which does not require training. The unsupervised approach does not yield much structure and differences in space do not show a clear clustering of GA or model error (Fig. 4d). In this regard, the spatial distribution of GA and error is more similar to a random model (series2signal model applied with randomly shuffled labels, Supplementary Fig. 8A). However, with the top-1 model’s embeddings, series2signal learns a more structured embedding that shows a clear spatial trend across GA. Applying a new, automated approach to identifying semantic clusters (see Methods), we also find a number of interesting clusters in the series2signal embedding. In particular, we find enrichment of depression-related variables in one cluster of the model embeddings, enrichment of comorbidities, and a healthy cluster measurements that indicate low stress and good sleep measurements. In contrast, the unsupervised learning approach only identifies missingness in one clinical variable, lower stress and less daytime drowsiness, and lower-than-expected enrichment of no past birth-control. Similarly, some of the random clusters are enriched for clinically irrelevant variables or variables confounded by missingness. This suggests that, especially with a larger cohort, series2signal may identify sub-populations of measurements that indicate not just GA but also, the co-occurrence of enrichment of clinically relevant variables and could provide for a fast visualization and summary of the health status of an pregnant individual over the past week, which may also be useful in treatment support or suggesting consultations for behavioral modifications that reduce the risk of prematurity.

## Discussion

We developed series2signal to analyze physical activity and sleep patterns during pregnancy using deep learning and found that deviations to activity and sleep, as measured with wearable devices, are associated with prematurity. As a proof-of-concept, we showed that the combination of wearables with machine learning and model analysis can be used to signal behaviors that heighten risk of preterm birth and suggest feasible interventions to reduce the likelihood of prematurity. To predict GA from wearables data, we used 1-week of activity data following 2305-GA measurements from a cohort of 1083 pregnant individuals and adapted a state-of-the art deep learning time-series classification architecture to monitor the progression of pregnancy via regression. We also developed a novel interpretability algorithm that integrates unsupervised clustering, model error analysis, feature attribution, and automated actigraphy analysis. series2signal allows for model interpretation with respect to activity, sleep, and static clinical variables. We found that our approach performs significantly better than 7 other machine learning methods in predicting GA (max *P* = 7.4e − 13, Mann-Whitney U; top-1 MAE, 7.52 weeks) and, generally, that with our deep learning analysis, we can measure the progression of pregnancy from wearables data (*P* < 0.001, compared to random models, Spearman’s).

Primarily, however, we found that our models’ prediction and its difference with the actual GA, an “actigraphy-GA,” is a robust signal of heightened risk of preterm birth. In particular, the incidence of preterm birth is a significantly different variable amongst error modes (*P* < 0.001, chi-squared). When our model predicts lower-than actual GA, there are 0.52 times as many preterm births than expected (*P* < 0.001, permutation test), while when our model predicts higher-than actual GA, there are 1.44 times as many preterm births as expected (*P* < 0.001, permutation test). In parsing out whether this model error is meaningful and robust, we found that model error (model-actual GA) is negatively correlated with interdaily stability (*P* = 2.7e − 4, Spearman’s), which indicates that our model assigns a more advanced GA for individuals with decreased circadian rhythmicity. Supporting this, our model attributes higher importance to sleep periods in predicting higher-than-actual GA, relative to lower-than-actual GA (*P* < 0.001, Mann-Whitney U). We found that our deep learning analysis with series2signal also yields semantically meaningful clusters through phenotyping and that our pre-trained models’ representations can be used for improved and lightweight prediction on several ancillary, but clinically relevant, tasks, broadly suggesting the utility of analyzing wearables data for signaling various health-related statuses during pregnancy. Broadly, we showed that combining prediction with interpretability allows us to robustly signal activity and sleep behaviors that reduce the likelihood of preterm birth, which supports the future development of clinical decision support and intervention through relatively inexpensive passive monitoring.

Preterm birth remains the largest cause of death amongst infants. While there may be biological risk factors in an appreciably large percentage of pregnancies that result in prematurity, behavioral components, such as sleep and physical activity habits, may also increase the risk of preterm birth^20^. We sought to demonstrate that inexpensive monitoring of pregnancy using wearables can signal deviations from physical activity and sleep patterns that are associated with full-term births with no complications. If this proof-of-concept were demonstrated, then wearables could be used to shift activity and sleep to mimic healthier individuals, perhaps reducing the incidence of prematurity and infant mortality with inexpensive monitoring.

Despite several biological studies, it has been hard to change the landscape of preterm birth from a global health perspective^20,21^. Studies of preterm birth that involve biological samples are expensive to conduct and their scale is limited. Wearable devices, on the other hand, can more easily distributed and have been shown to be useful in other fields^7^. Thus, if we are able to use computational techniques to identify patients at heightened risk for preterm birth, we can target sample collection and improve the efficiency with which we more deeply understand the biological versus environmental underpinnings of preterm birth. Indeed, series2signal identifies measurements that are associated with higher prevalence of preterm birth when the model thinks the measurement comes from an “older” pregnancy *and* a lower prevalence of preterm birth when the model thinks the measurement comes from a “younger” pregnancy (Fig. 2). Using this information, we can use wearable devices to follow the trajectories of pregnant individuals, indicate which phenogroup they may belong to and then select a sub-sample for more detailed followup. This would especially improve enrollment in LMICs, where access to biological samples is more difficult.

Despite the advances in our study, prediction is not enough: while monitoring the progression of pregnancy and sleep and activity behaviors may signal to clinicians that heightened attention is in order, longer-term, we hope that our study can also help in the development of effective intervention and correction. series2signal may be useful in suggesting inexpensive interventions. For example, one significant difference amongst error groups is in the extent of weekend activity (Supplementary Fig. 3A). This suggests that series2signal can identify when a pregnant individuals’ past week of activity emulates a higher-risk form of activity and sleep pattern, given the GA. Signaling poor sleep-quality or activity that progresses to the point of heightened risk may enable clinicians to alert pregnant individuals about their sleep status in the past week and help individuals prioritize proper sleep hygiene to mitigate the risk of prematurity and neonatal complications. Thus, while predicting the progression of pregnancy was the main task in the paper, we view that series2signal will be most helpful in developing relatively inexpensive interventions in the future, since the combination of LMP and ultrasound remain more accurate than wearables in determining GA (Fig. 1f).

Previous studies have shown that heightened stress portends an increased likelihood of prematurity. Outside of pregnancy, it is broadly known that physical activity and sleep are amongst the strongest modulators of stress and physiological inflammation. This suggests that connecting stress to prematurity can effectively be studied with wearables; however, there remains a preponderance of processing such data and converting it into actionable insight. We developed series2signal to fill that gap. In particular, wearable devices allow us to objectively quantify physical activity and sleep, and serve as a proxy for stress measurement. However, they produce a large amount of data and their analysis is not trivial. Here, we build a new machine learning pipeline that takes 1 week of raw actigraphy data and converts it into GA, namely, how far along the pregnancy is. Then, relative to the actual GA (measurable through LMP or ultrasound), we show that deviations from normal activity and sleep is associated with poor pregnancy outcomes (Figs. 2,3). This gives us an opportunity to use an accessible, non-pharmaceutical intervention to modulate the biological mechanisms that drive pregnancy outcomes or prevent negative outcomes. series2signal will also allow us to further investigate pathways affected by altering sleep and activity, including inflammatory signalling and HPA axis activation.

There are a number of limitations to this work. In particular, small data will plague the generalizability of our conclusions (this cohort is comprised of *N* = 1083 patients, with *N* = 2305 measurement-GA pairs). However, we developed new algorithms to reduce over-fitting and improve generalization to a held-out test set (see data augmentation in the Methods). Furthermore, our goal was to conceptually demonstrate that wearable device data can be used to *indicate* deviations from physical activity and sleep associated with clinically relevant high-risk groups, such as preterm birth. While the study is small on the scale of obstetrics, our cohort population has 706 (56%) African American individuals. Black, pregnant individuals are often under-representented in obstetric studies^22,23^. Our study overcomes this barrier, while maintaining sufficient numbers for training accurate machine learning models without over-fitting. Other limitations include the simplicity of sleep detection. More sophisticated methods may reveal more subtle and preventable disruptions in sleep that increase a patient’s risk for PTB. Here, we rely on a previously developed method that builds on count-based thresholds for sleep detection^24^. Though series2signal establishes the potential usefulness of wearable devices in understanding the progression of pregnancy and its relation to some clinical outcomes, including PTB, we have not explicitly disentangled our “clock” of pregnancy into independent sleep and physical activity disruptions, which may offer fruitful further study in combination with improved sleep annotations. In addition, given the size of our network, the model is still best able to compare pregnancies within this cohort. The generalizability to wearable device data collected in other clinical settings, e.g., not in the US, may be more limited. Lastly, we automatically filter measurements for which patients did not wear the device, or remove it for an extended period of time during the week prior to their GA measurement. In the future, with larger data, we may improve data quality screening and collection by relying on data that doesn’t span entire weeks. Furthermore, in future work, we may benefit from harnessing activity data from non-pregnant individuals. This can be used to score activity and sleep quality and be drawn upon as source data to generate new time series, augmenting our target data with activities typical of non-pregnant individuals, which may amplify accuracy with respect to later gestational ages^25^. We may also improve our augmentation scheme to include latent space augmentations rather than time-domain and frequency-domain augmentations, which might facilitate comparison of actual and generated activity traces and suggest more precise activities and sleep behaviors that can reduce the risk for any given individual on a more personal level, relative to the comparison against groups, that we do here. Finally, an exciting future direction of work remains the targeted collection of biological samples that will allow us to pair behavioral modulation with physiological or pharmacologic intervention to reduce the incidence of preterm birth.

## Methods

### Cohort and data description

#### Human subject recruitment and data collection

Institutional Review Board approval was obtained from the Washington University School of Medicine Human Research Protection Office. Patients from the Washington University School of Medicine and local community who were planning pregnancy were recruited to the study. Pregnant individuals were included in this study if they were at least 18 years of age, preparing to conceive, and willing to wear an actigraphy monitor throughout their pregnancy. Patients that had multiple gestations, underwent in vitro fertilization, had a uterine anomaly, or had used sedatives were excluded. Patients who signed informed consent documents wore activity monitors throughout their pregnancy until delivery. Gestational age was measured at enrollment by last menstrual period (LMP) and < 14 weeks ultrasound (*N* = 1189(49.6%)), LMP (*N* = 651(29.0%)), < 14 weeks ultrasound (*N* = 413(18.8%)), and > 14 weeks ultrasound (*N* = 38(1.9%)). From these, regular follow-ups were used to assign a gestational age label to downloaded actigraphy data.

We summarize our cohort using the PECOT framework (see Supplementary Fig. 1). Population: 1260 individuals were enrolled if they had a singleton pregnancy ≤ 20-weeks gestation during the study period of January 2017 to January 2020. Inclusion criteria were that the individual (A) planned to deliver at Barnes-Jewish Hospital, (B) were English-speaking, and (C) were at least 18 years old. Exclusion criteria were (a) prior incarceration, (b) conception via in vitro fertilization, and (c) diagnosis of major fetal anomaly that affected gestational age at delivery by attending physician. Exposure: Study participants were seen at study visits longitudinally to obtain data and samples in each trimester. All study participants were given validated questionnaires about sleep habits and lifestyle and wore actigraphy devices (Motionwatch8, CamNTech, United Kingdom) continuously (24/7) for two-week time periods immediately following their first, second, and third trimester study visits to assess circadian rhythms longitudinally throughout pregnancy and delivery. Control: All participants in this study received the exposure, as well as incentives and reminders and fully charged, 90d actigraphy devices to ensure full data collection. Retrospective sub-groups were created from these participants based on associated clinical data. Outcomes: A number of pregnancy characteristics and outcomes were tracked (see Table 1). In particular, outcomes of interest included pregnancy complications such as gestational hypertension or preeclampsia, and delivery outcomes such as live births, stillbirths, pregnancy losses, and type of delivery procedure. A primary outcome of interest was whether or not participants with a live birth delivered at term or delivered preterm. Neonatal complications were also tracked, including their birthweight relative to their GA, and whether they were admitted to the neonatal intensive care unit. Timeline: Study participants were seen at study visits throughout their pregnancy and at delivery and aligned with routine medical care. Study visits fell within each of the trimesters, at which point patients were given questionnaires and devices that were returned at the follow-up visit or during delivery, with incentives and research staff reminding patients longitudinally to complete all study data. Device data from preceding trimesters were downloaded at the subsequent follow-up visit. Further details on this cohort are available from our prior publication^26^.

### series2signal algorithm

The series2signal algorithm is described in detail in the Supplementary Material. Briefly, it consists of 5 modules:

1. Data pre-processing and automatic sleep-wake annotation.
2. Model training and data augmentation.
3. Model error analysis.
4. Model interpretability using gradient-based feature attribution and a new feature association score for mixed tabular data of continuous and categorical variables.
5. Model utility in-terms of predictability from concise representations and automatic phenotyping.

#### Data pre-processing

*Data cleaning*: To align the actigraphy data, we arbitrarily chose to select 12 AM as a starting point for each tensor. In the worst case, for measurements starting at 12:01 AM, this would amount to a full day of zero-padded data. To avoid encountering this problem, we threw out the first day of measurement entirely for all data. Thus, strictly speaking, series2signal requires 8d of recording. Recordings are truncated and, if trailing padding is required, they are excluded from analysis, as a way to filter out for measurement-GA pairs without a full week of recording. Raw actigraphy data was read in from device files (.mdn files) using pyActigraphy^8^ and paired with the appropriate metadata, collected in the clinic, using custom scripts. Data that had any mismatch between raw data filename and metadata record identifier, no light intensity information, missing data from minute-to-minute, or less than 1d of recording were excluded from analysis to ensure quality input to series2signal. Metadata was also cleaned and pre-processed to give categorical encodings to indicate missingness and to mean impute missing continuous data. Specification of each variables’ pre-processing is stored in the GitHub repository for series2signal and for different metadata, providing a similarly structured dictionary mapping variables as keys to a set of transformations can be custom-designed by users for their application in a text file, which series2signal can read. The top-1 model was trained using patient data from *N* = 658 individuals (with *N* = 1399 samples), validated on *N* = 100 individuals (with *N* = 216 samples), and tested and evaluated on *N* = 325 individuals (with *N* = 690 samples). Data splits for all trials were separated by patient so that no measurement from the same patient leaked into different splits. From a cohort of *N* = 1260 patients with full outcomes data, we filtered out individuals whose devices did not have light data, had corrupt data, or less than 1wk of recording post GA-measurement, yielding a total dataset size of *N* = 1083 patients with *N* = 2305 samples.

*Data transformation and normalization*: Raw count data (sum of accelerometer counts over a 1-min interval and light intensity measurement in lux) was log-pseudocount transformed (${\log }_{10}(x+1.0)$), where *x* represents the filtered actigraphy sequence data, which is standard for the field of actigraphy analysis^27^. We represent the data as a 2 dimensional time-series of length *L* = 10,080 min (1 week), leading to an actigraphy representation of shape *N* × 2 × 10,080. This sequence, post-log pseudocount transformation, served as input to the series2signal model. We also add a number of standard non-parametric analyses by writing a wrapper for pyActigraphy in our codebase, which allowed us to include IS, IV, RA, ISm, IVm, and other summary metrics of actigraphy data as part of each sample’s metadata^8^.

*Automatic sleep detection*: Previous studies have examined different methods and established benchmarks for sleep vs. wake detection based on paired polysomnography with actigraphy data^24^. Their results show that traditional algorithms for sleep detection with re-scoring rules work well for sleep vs. wake detection whereas deep learning algorithms perform better without re-scoring rules^24^. Based on these results, we take the best traditional algorithm, due to the marginal improvement of more complicated methods, applying the Oakley method after data cleaning and raw mtn file reading (with Oakley’s threshold *θ* = 80) with pyActigraphy^24^.

### series2signal machine learning pipeline – data and prediction

#### ResNet-inspired series2signal deep learning model

In the field of time-series classification, deep learning has not consistently outperformed feature engineering and classical machine learning approaches and non-deep learning approaches do not scale well; for example, the non-DL state-of-the art with *N* = 1500 samples and sequence length *L* = 46 takes more than 8 days to learn^11^. Our dataset is considerably larger, and therefore, many algorithms from the time-series classification field are not applicable. Recently, a deep learning architecture based on ResNet recently outperformed more traditional time-series classification algorithms^11^. We adapted this time-series classification architecture for time-series regression. We also modified the internal batch normalization to a layer normalization within the blocks, finding that this achieved better performance. We also add a novel training scheme involving data augmentation in order to improve this ensemble of deep convolutional neural networks generalization and reduce over-fitting to a small training set size. Lastly, we add a non-linear prediction block to allow for us to capture non-linear embeddings of any set of input actigraphy data, which we reasoned would be useful for phenotyping, data visualization, and more lightweight classification and regression applications on auxiliary tasks. Additional details are provided in the supplement.

*Benchmarking series2signal model architecture with other ML methods*: To compare and justify the use of a more complicated ensemble of CNNs for analyzing actigraphy data, we compared our series2signal model to various convolutional neural networks (VGG-1D and a simple CNN of *d* = 256 hidden units) and recurrent neural networks (bi-LSTM and bidirectional GRU, each of 3 layers and *d* = 64 hidden units) using custom implementations in PyTorch v1.9.0, available on our GitHub. We also compared our series2signal model to standard machine learning methods using scikit-learn^28^ (kNN with *k* = 5 neighbors, elastic net logistic regression with $\frac{{\lambda }_{1}}{{\lambda }_{2}}=0.1$, and RandomForest with default parameters) and *k* = 5 fold cross-validation, split on patient groups and wrote wrappers for sktime’s adaptations of RandomForest and kNN algorithms for time-series^29^. Lastly, we compared our series2signal model to a popular gradient boosting method with default parameters using lightgbm^30^.

#### series2signal model training and implementation

*Model training*: To adapt the InceptionTime time-series classification model to a regression task on a small data set, we added a *λ*_1_ penalty in addition to the loss function, in addition to the standard weight decay on MSELoss. We found that a small penalty of *λ*_1_ = 1e − 6 and *λ*_2_ = 0.001 minimized error in a hyperparameter search. To train the series2signal model, we used Adam optimization^31^ and used PyTorch’s implementation of reducing the learning rate once our loss function, ${{{{{L}}}}}_{m}={\lambda }_{2}\cdot \frac{1}{m}\cdot \mathop{\sum }\nolimits_{i = 1}^{m}{(y-\hat{y})}^{2}+{\lambda }_{1}\cdot \mathop{\sum }\nolimits_{i = 1}^{m}| y-\hat{y}|$ reached a plateau of patience = 10 epochs^32^ for mini-batches of size *m* and for various labels, *y*, including GA. Models were evaluated on a validation set after a minimum of 200 epochs, and then passed to the learning rate scheduler.

*Data augmentation*: We used PyTorch’s data loaders to load actigraphy data into tensors during optimization. Once a mini-batch of samples was loaded, we then applied a new augmentation procedure, which we optimized for minimizing error (see Supplementary details; Supplementary Table 1). To select data augmentations for the architectures we attempted, we rely on a categorization scheme used in the UCR dataset^33^, relating our time-series data most closely to the ECG data category (selection is the same if we rely on the motion category recommendations). With this categorization, we apply the recommendations from a 2021 survey of data augmentation techniques for time-series classification^34^. To simplify their suggestions, we took the top-5 methods after a union of methods for CNN-based architectures and RNN-based architectures and then took the intersection of the two ( ⋃ (VGG, ResNet) ⋂ ⋃ (LSTM, BLSTM)). This suggested that we use scaling, rotation, none, window warping, and jittering augmentations. For window warping, we selected a random window that is 10% of the time-series length, *T*, and which warps the time dimension by a factor of 2 or $\frac{1}{2}$, following^35^. For jittering, noise is added to the time-series after drawing from the normal distribution, ${{{{N}}}}(\mu ,{\sigma }^{2})$ and we set *μ* = 0 and *σ* = 0.03, following^36^. For scaling, *α* was drawn from ${{{{N}}}}(\mu ,{\sigma }^{2})$ and the time-series was modified as follows, ${{{\bf{{x}}}^{{\prime} }=\; \alpha {{{{\bf{x}}}}}_{1},\; \ldots \alpha {{{{\bf{x}}}}}_{{{{\bf{t}}}}}}}$ where the random scalers, *α* is drawn from a Gaussian with *μ* = 1 and *σ* = 0.2 per time-series, as in ref. ^36^. Window slicing was performed to crop each time-series to 90% of the original sequence length, *T*, starting the window at a random *t* ∈ *T* and interpolating the cropped time-series back to the original length, *T*, as in ref. ^35^. However, rotation actually reduces accuracy across datasets, so we omit this transformation^34^, replacing it with slicing, which is shown to perform well across all UCR datasets and has low computational requirements. We abbreviate these transformations N (None), Sc (scaling), J (jittering), W (window warping), and slicing (Sl). To implement these augmentations during training, we randomly select a single transformation per mini-batch and per epoch, finding that the XXX is better. For inference, we query the trained network for each test sample and average the output across transformations. To our knowledge, this approach is novel with respect to data augmentation for time-series because it combines data augmentation methods during training, uses a majority-voting or average inference method, and evaluates whether transformations should be applied at the top of the mini-batch or epoch^37^. To optimize data augmentation in series2signal algorithm, four different schemes were tested to select from a set of time-series data augmentation functions during model training; either a random augmentation was selected for the whole optimization process (randaug), all augmentations in random order (allaug), or a different iteration of each of these per epoch. Augmentations were selected based on a recent meta review^12^. Supplementary Table 1 shows the data augmentation schemes we implemented during training of our networks, as well as the schematic for evaluation, and results showing the superiority of selecting a random augmentation per epoch from our set of augmentation filters results in the best performance with respect to the primary task.

### Evaluation

Details of the series2signal modules for knowledge discovery and model interpretability are provided in the supplement. Briefly, error analysis modules comprise a new correlation network methods and sub-group analyses to identify error modes in model output and performance. Similar statistical approaches are then applied to gradient-based feature attribution methods to automate series2signal model interpretation with respect to identified key variable and observation groups.

#### Metrics used in benchmarking for series2signal algorithmic development

For balanced accuracy in multi-class classification, we follow the approach of ref. ^38^, computing the average of sensitivity and specificity per class, then averaging over the total number of classes. This adjusted balanced accuracy allows us to evaluate classifiers even where there is class imbalance using the area under the precision-recall curve. We evaluate regression tasks via Spearman’s *ρ*, such that higher correlation between model output and label indicates a better regressor. We also compute the mean absolute error, and the mean absolute percentage error (MAPE, to compare to metrics on a range of 0 to 1). To evaluate classifiers, we use AU-PRC and an adjusted AU-PRC metric, that re-scales the AU-PRC after subtracting the AU-PRC of a random classifier with the same labels as the inference set (see Supplement for details). We also compute a balanced accuracy score using sklearn^28^. We benchmark to other machine learning pipelines on the basis of these metrics per task and define tasks as classification or regression based on the data type, where all categorical metadata variables are treated as classification tasks, and all continuous metadata variables are assigned as regression targets. To implement classical machine learning algorithms, we wrote wrappers for sktime, allowing us to use TimeSeriesForest, an adaptation to the widely-used RandomForest algorithm for time-series, and kNN-TimeSeries, a dynamic time warping modification to kNNRegressor^29^. To compare clustering, we apply the same pipeline but develop a custom unsupervised learning approach to contrast series2signal’s supervised learning embeddings. Internal parts of all algorithms also used these metrics to inform design choices.

#### Statistical analysis

To summarize data, if data were normal (by D’Agostino and Pearson’s test^39^) then mean and standard deviation were shown, otherwise median and inter-quartile range was shown. Categorical variables were assessed for differences across groups by Fisher’s exact test and continuous variables were compared across groups by a Kruskal-Wallis test. In head-to-head comparisons for continuous data, either Spearman’s *ρ* or Mann-Whitney U tests were used and categorical, when ordinal and compared with other categorical data, was assessed by the Goodman Kruskal Gamma. Categorical-continuous variables were compared by logistic regression, using the categorical variable as binary or multi-class classification and micro-averaging performance per class after adjusting for class imbalance via SMOTE^40^. *χ*^2^ tests were used to compute observed and expected ratios for the prevalence of various metadata variables. Permutation tests were performed for derived metrics for at least *n* = 1000 iterations and after the null distribution was created by random sampling over groups and preserving group ratios, a Mann-Whitney U test was performed to statistically compare the difference between observed and null metrics. All P-values were corrected for multiple comparisons by applying the Bonferonni correction method (*p*_*corrected*_ = p*n_*comparisons*_) where appropriate, unless specified. All statistical analyses were performed with scipy, numpy, and scikit-learn in Python v3.8.12^28,41,42^.

### Reporting summary

Further information on research design is available in the Nature Research Reporting Summary linked to this article.

### Supplementary information

Supplemental Information

Reporting Summary

## Data Availability

Raw wearables data, processed wearables data, and the static analysis source code used in this study are available from https://nalab.stanford.edu/series2signal-gestational-age-clock-for-pregnancy-monitoring/.

## Code availability

Our series2signal software is available for use on custom datasets through GitHub at https://github.com/nealgravindra/wearables under an MIT License. Models can be trained and post-hoc inferences can be generated using our new series2signal approach with existing PyTorch models and either CPU or GPU versions. All analyses and results presented in this paper are also available on our GitHub repo for reproducibility and the code used to generate each figure and table is specified in a reproducibility table within the same GitHub repository (see here).
