# Supplementary material for: Deep representation learning identifies associations between physical activity and sleep patterns during pregnancy and prematurity
Source: NPJ Digit Med. 2023 Sep 28;6:171. doi: 10.1038/s41746-023-00911-x (PMC10539360; doi:10.1038/s41746-023-00911-x)
Supplement: Supplementary file 1 — Supplemental Information [file 41746_2023_911_MOESM1_ESM.pdf]

Supplemental Information

Methods

series2signal algorithm

series2signal consists of 5 modules:

1. Data pre-processing and automatic sleep-wake annotation
2. Model training and data augmentation
3. Model error analysis
4. Model interpretability using gradient-based feature attribution and a new feature association score for mixed tabular data of continuous and categorical variables
5. Model utility in-terms of predictability from concise representations and automatic phenotyping

In order to model 1wk of actigraphy data, which consists of 2-dimensional time-series data of length $L = 10,080$, given the 60s integration of counts, we make a number of adaptations to a deep learning architecture, which allows us to turn multi-variate time-series data from actigraphy devices worn by pregnant individuals into a single-output, namely the model GA (Figure 1D). To accomplish this, we adapted a state-of-the-art deep learning model for time-series classification tasks to regression, added a shortcut layer and improved upon the training algorithm with data augmentation pertinent to time-series data with long ($L > 1000$) sequence length. The machine learning pipeline is summarized in Figure 1E. From raw data, series2signal pre-processes actigraphy accelerometry and light-intensity data, optimally selects time-series data augmentations to improve performance on the primary task, and trains a 1D-CNN-based deep learning model to output GA from 1wk of wearables device data. Post-training, a number of inferences can be made on top of the model to provide additional insight into an individuals' pregnancy.

We also automate a model error analysis, in which we compare readily available ground-truth GA measurements with the prediction from the model to identify groups that we can associate with other metadata, including tabular data from clinical records (EHR data or metadata).

To more deeply investigate at what time of day or week leads to deviations of physical activity from normal, series2signal includes a model interpretation module, which allows us to ascribe which periods of the past week contribute to model predictions. This, combined with similar automated sub-group discovery and a new function for tabular association network discovery, will enable individuals to distinguish whether periods of activity during the week or weekday or during sleep versus wake contribute to deviation using series2signal.

Given the extent of metadata often associated with each individual patient, we also demonstrate that the machine learning pipeline of series2signal can yield clinically useful visualizations of an individual at any point during their pregnancy relative to the training cohort and the embeddings can also be used in light-weight ML tools to indicate various clinical indications. Based on this overall analytical pipeline and collection of sub-modules, individual variables of interest or analyses can be selected for additional development or customization, depending on the setting, as we provide the series2signal as a Python package.

The modules in series2signal can be used independently of one another but the model error analysis, model interpretability, and model utility components require a pre-trained model. From step 1 through step 5, series2signal produces a GA prediction's and analyzes the utility of that predictions difference with available GA to signal deviations to sleep and activity that may or may not indicate that the pregnancy is at heightened risk for prematurity. series2signal can be lightly adapted to be compatible with any multi-variate time-series dataset and other wearables data, although the data pre-processing model is designed to be compatible with actigraphy data.

series2signal model

Details of series2signal model architecture: To select the optimal architecture for series2signal, we performed grid search hyperparameter optimization and selected the set of hyperparameters that achieved the lowest MAE on the primary task (GA prediction) on a validation set. Hyperparameters included batch size, the maximum number of epochs, learning rate scheduler type, number of epochs for patience, minimum number of epochs for training, dimensionality of bottleneck, filter sizes for the Inception blocks, and the number of filters in each block. We also experimented with different sizes and dimensionality of the prediction blocks. series2signal's final architecture consists of 9 inception blocks, a bottleneck layer with size $d = 1$, kernel sizes of $k_1 = 96$, $k_2 = 32$, $k_3 = 4$, with 32 filters, and residual connections. Additional details are provided in the model code for the series2signal method on GitHub.

series2signal modules for knowledge discovery: model interpretability

Error analysis

The details of the series2signal modules for this cohort and broader algorithmic details, which are relevant to other applications of wearables analysis, are provided here. To investigate this cohort, we used series2signal's error analysis module to first identify error trends. The best performing model (top-1 or best series2signal), as evaluated on the held out test set, was saved and its model parameters stored. Then, in comparison to the actual GA, we used the error (model minus actual GA) to identify error groups. Error groups were selected by picking $k = 3$ and maximizing balance between the two primary error modes closes to the mean absolute error performance on the test set for the best model, yielding an over/under threshold of 10wks to delineate between error-free and higher- or lower-than-actual error groups. Once we had identified these error groups and thresholds, we were able to compare the label with model output by querying the model for each sample in the cohort, assigning each measurement-GA pair to one of three error groups. Once we had these groups, we compared this variable across the cohort with all available metadata, which included medical histories, clinically collected information, and surveys around activity, stress, social determinants of health, and depression. We designed a custom tabular correlation network function to provide an association score between mixed categorical and continuous variables in tabular data. To come up with an association score that represents the strength of correlation, we first considered the variable type. For two ordinal variables, we used the absolute value of the Goodman-Kruskal γ, which tests for a monotonic relationship using concordant and discordant pairs. To score the magnitude of association between two continuous variables, we use the absolute value of Spearman's ρ. To score the association between continuous and categorical variables, we apply the synthetic minority over-sampling technique [1] to create balanced classes (multi-class in the case that there are multiple values for a categorical variable, via one-hot-encoding). Then, we use elastic net logistic regression with $k = 5$ fold cross-validation, split on patients, and estimate the macro-average AU-ROC between classes to get an association score. By pairwise comparison between values in the metadata, we are able to construct a correlation network based on the cohort, where the edge weight in the adjacency matrix is given this association score. For each metadata variable, we can then use χ^2 and Mann-Whitney U tests to compare the categorical or continuous variables' differences across error groups to create a graph where the node size is proportional to the variables' difference across error groups. We can also create a graph where the node size is equivalent to the predictability with a lightweight, $k = 5$ kNN regressor or classifier based on activity counts alone to indicate the predictability of that particular metadata variable. To validate any identified differences in error group with respect to a clinically relevant variable, e.g., whether the pregnancy resulted in a preterm birth, we performed permutation testing, using the *chi* statistic, which allows us to compare the expected value in cross-tabulated data, namely, what the values of each variable would be in each of the error groups if there were no association between error group and a particular variable [2]. For categorical to continuous variables, we relied on a $\log_2$ fold-change between error groups as a metric. For sampling, we sampled only from the test set, and created a null distribution by randomizing the error group label. After $n = 1000$ iterations, we constructed observed and null distributions of the metric for a particular variable, and compared, by independent t-test, these distributions to validate or invalidate our finding in the training set. Correcting p-values for multiple comparisons then allowed us to identify significant differences in clinically important variables between error groups.

series2signal model interpretation

To address why series2signal models provide their indication of the point of progression in a particular pregnancy based on actigraphy data, we rely on gradient-based feature attribution methods. In particular, we use a variant of integrated gradients, which creates linear interpolations of an input, relative to a baseline of 0 log-pseudocount activity and calculates the gradients for each interpolant, to measure the relationship between changes to a feature (log-pseudocount activity at a particular time) and changes in the model's predictions, such that higher attribution values are given to features whose variation affects model output, scaled to the input [3, 4]. To smooth this variance output per feature and input sample, we average gradients and add noise to $n = 10$ inputs [5]. We adapt the `PathExplain` implementation of feature attribution for our series2signal model, which allows us to, for each input activity trace, compute a feature importance score (absolute value of feature attribution) per point in time (Figure 2A). With these feature importance scores, we can use the actigraphy's time-indexing to create feature groups by time-of-day or time-of-week. We can also use the automatic sleep annotation to group features. Via sampling from these periods per patient measurement, we can apply the association scoring used in our custom tabular correlation network, described above. This allowed us to associate feature importance and model error with metadata variables and time-of-day and -week to explain why deviations to sleep and activity lead to useful predictions, when compared to the actual GA.

series2signal semantic clustering for phenotyping and predictability of non-linear series2signal embeddings

From the series2signal top-1 model, we extracted the hidden representations for all samples (by inference and querying the best model per sample) by outputting the GA in addition to the trained model's learned and compact representation of the actigraphy input, prior to the non-linear prediction blocks. These represent the model's learned representation of the actigraphy data. To determine whether these representations were semantically meaningful and useful, we performed time-series clustering based on these embeddings and identified and annotated clusters by a novel statistical hypothesis testing procedure. To compare this time-series phenotyping, we also developed a pipeline for unsupervised clustering of time-series by computing pairwise distances between time-series samples using dynamic time warping, as implemented with `fastdtw` [6]. We computed the pairwise distance between actigraphy samples based on the model embeddings using a high-dimensional distance metric, the Manhattan block distance. With each of these two pairwise relationships, we applied an affine transform (Gaussian radial basis function kernel) to create a weighted adjacency matrix, which allowed us to perform dimensionality reduction on the higher-dimensional representations (pre-processed actigraphy data in the unsupervised version) and perform graph clustering based on the Leiden algorithm for community detection and optimization of modularity [7]. Once clusters were identified in each group, we performed either Kruskal-Walis or Mann-Whitney U tests to each metadata variable, grouping by each cluster versus the remaining, to annotate each cluster. We removed any non-significant differences and selected the value for that cluster with the highest p-value (after correcting for multiple comparisons) to assign an annotation for a particular cluster. We compared the learned model representations to representations learned by a model with shuffled labels (see Supplement) to ensure the utility of supervised learning representations. To additionally assess the utility of series2signal modeling, we also, per metadata variable, compared kNN regressors or classifiers per task (defined by metadata variables) based on input as pre-processed actigraphy data ("raw") against the model embeddings, which is considerably lighter weight and faster in performing these auxiliary tasks.

Supplemental Figures and Tables

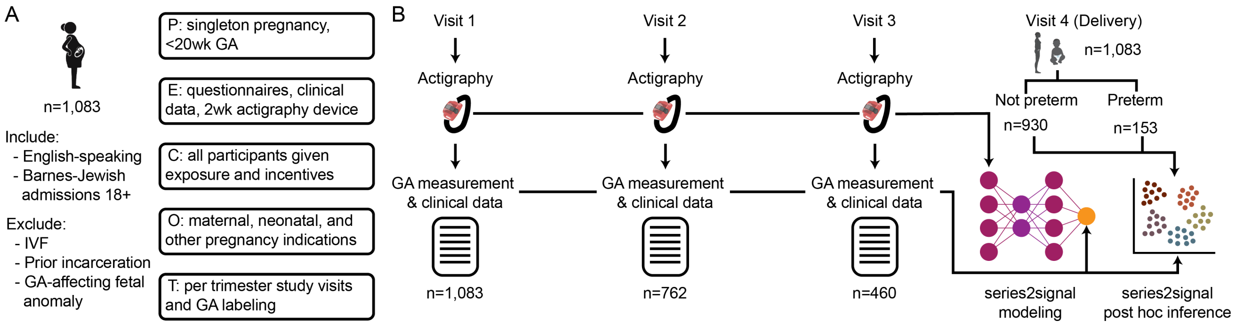

Supplementary Figure 1: Cohort diagram and clinical study workflow. (**A**) Cohort inclusion and exclusion criteria presented in the population, exposure, control, outcomes, treatment (PECOT) framework. IVF=*in vitro* fertilization. (**B**) Flow of wearable device measurements and labels between clinical study and *series2signal* computational analysis software and machine learning system reported in this study.

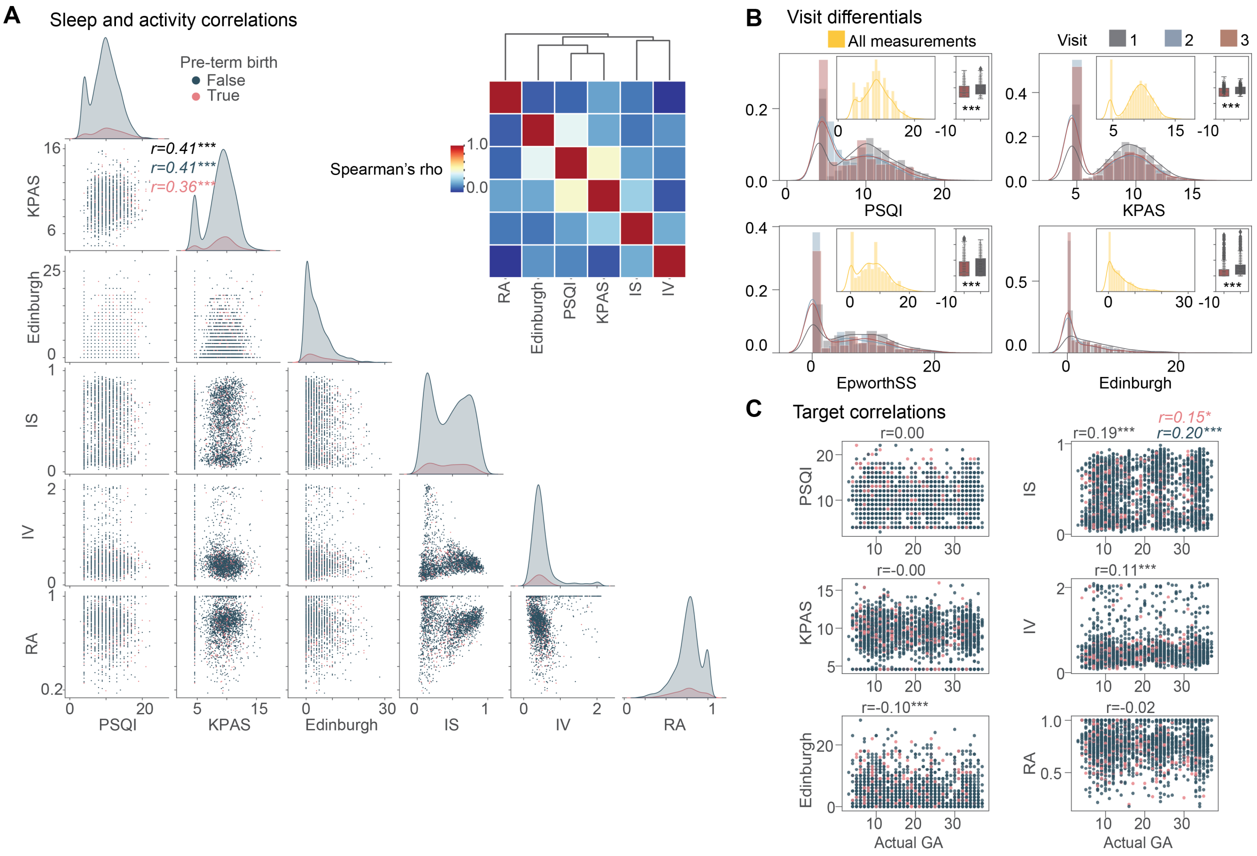

Supplementary Figure 2: Standard analyses fail to identify elevated risk prematurity. (**A**) Spearman's ρ correlation of standard non-parametric acitivty- and sleep-related variables in traditional actigraphy analysis and aggregation of survey result indicating depression, sleep-quality, and activity. Inset shows magnitude of ρ for indicated variables, where column and row annotations match. Box plots show median and first and third quartiles with outliers as 1.5 times IQR. Comparison of continuous variables is by Mann-Whitney U test. (**B**) Distribution of activity, sleep, stress, and depression variables in visits 1, 2, and 3, representing progression across pregnancy. (**C**) Spearman's ρ correlation of activity-related variables with GA.

	MAE (wks)	Spearman's ρ	P vs. randaugperepoch
randaug	8.00 (0.17)	0.35*** (0.02)	1.89e-01
allaug	8.31 (0.29)	0.36*** (0.04)	1.53e-02*
randaugperepoch	7.86 (0.25)	0.35*** (0.02)	-
allaugperepoch	8.16 (0.53)	0.41*** (0.03)	1.89e-01

Supplementary Table 1: Optimizing data augmentation in actigraphy2GA algorithm. Four different schemes were tested to select from a set of time-series data augmentation during model training; either a random augmentation was selected for the whole optimization process (randaug), all augmentations in random order (allaug), or a different itteration of each of these per epoch. All comparisons were pairwise by MannWhitney U test without correction for multiple hypothesis testingr with the minimum-achieved MAE, randaugperepoch.

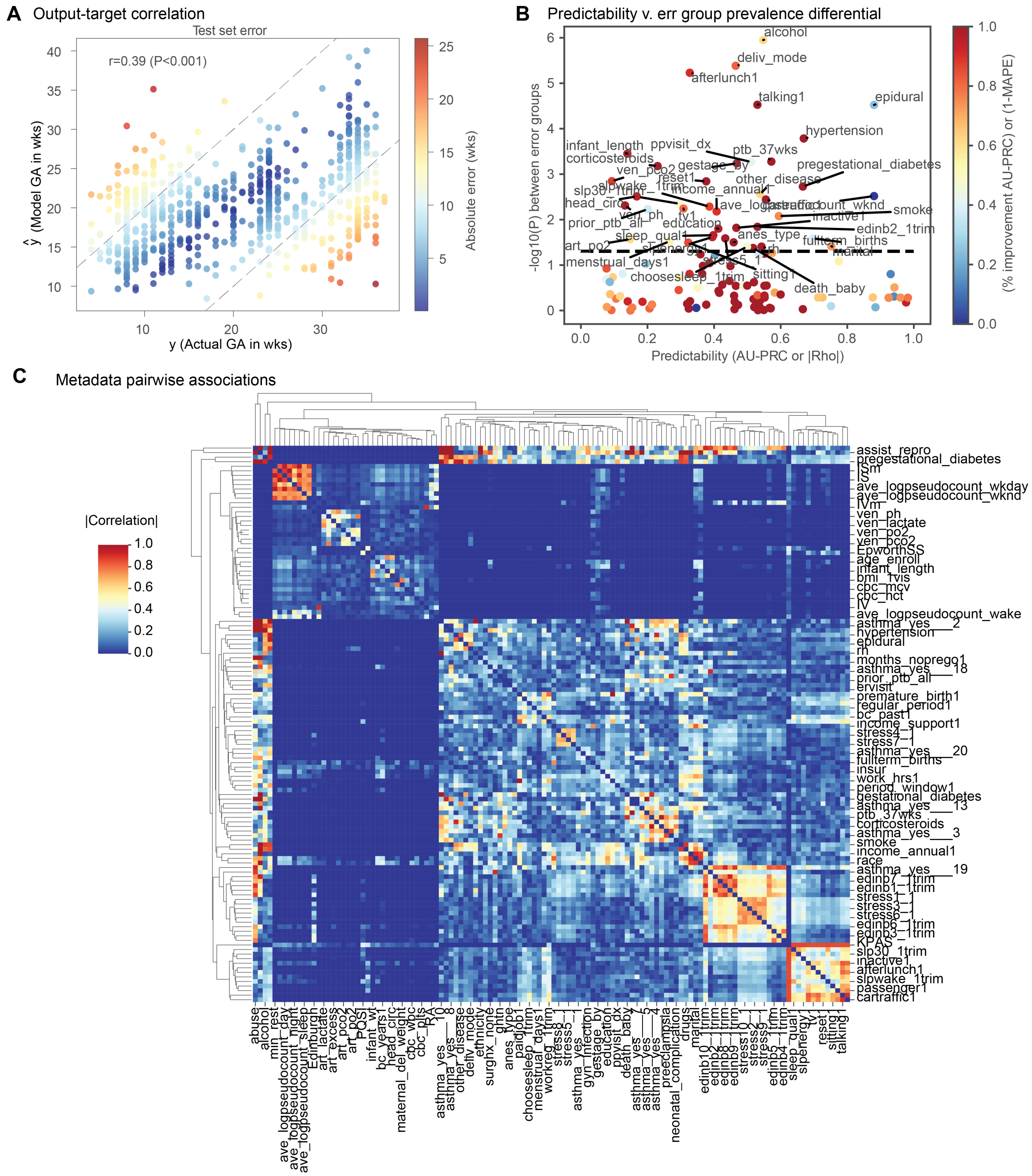

Supplementary Figure 3: Top-1 series2signal model error analysis identifies error groups and trends that differ in each. (**A**) Model predictions versus actual GA in the test set showing number of measurement-GA pairs in each error group. (**B**) Kruskal-Wallis or Mann-Whitney U multiple comparisons corrected P value versus predictability, defined us AU-PRC or Spearman's ρ with annotations for significantly different metadata variables across error groups (significance threshold, dashed line). (**C**) Correlation network showing the absolute value of the association between pairs of metadata variables (magnitude defined in main text Methods). Annotation in columns and rows match but are denoted as every other in each for readability.

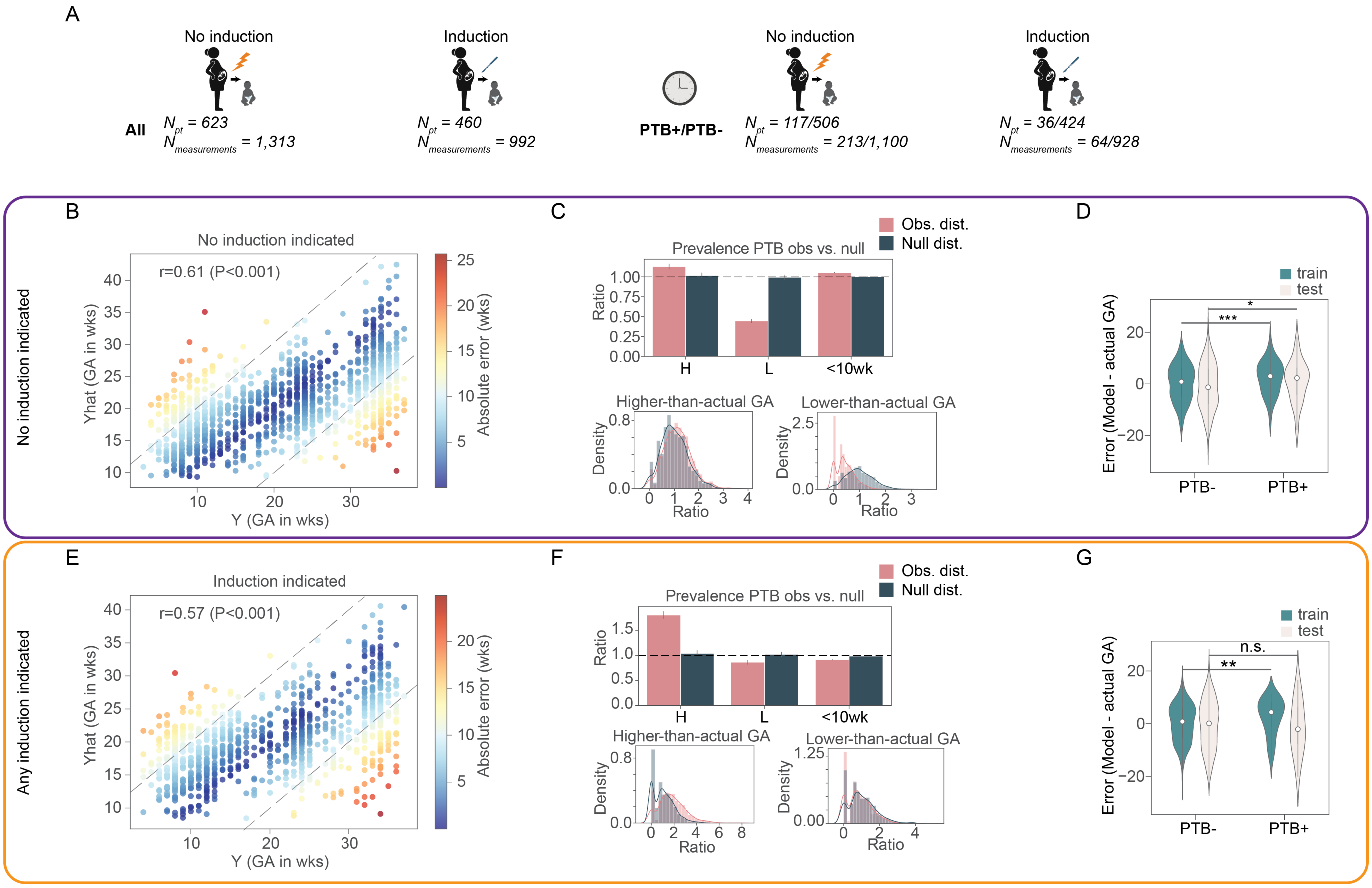

Supplementary Figure 4: Sensitivity analysis of differential PTB prevalence in error groups for births with any indication of induction vs. not. (**A**) Number of patients in cohort with and without any indication of induction and the prevalence of preterm birth in each subset. (**B** - **D**) Error analysis of series2signal model output in the no-induction indicated group. (**E** - **G**) Error analysis of series2signal model output in the group with induction indicated for any reason. (**B**/**E**) Correlation of actual vs. predicted GA. (**C**/**F**) Permutation test results showing odds ratio (bar-plot) of prevalence of PTB in H=higher-than-actual, L=lower-than-actual, and low-error groups and the distribution of each trials' OR (density plots) in observed and null distributions (color). (**D**/**G**) Model performance in train and test splits with Mann-Whitney-U test comparing magnitude of error between PTB-/PTB+ groups. Error bars indicate standard deviation for barplots and violinplots display distribution median and IQR.

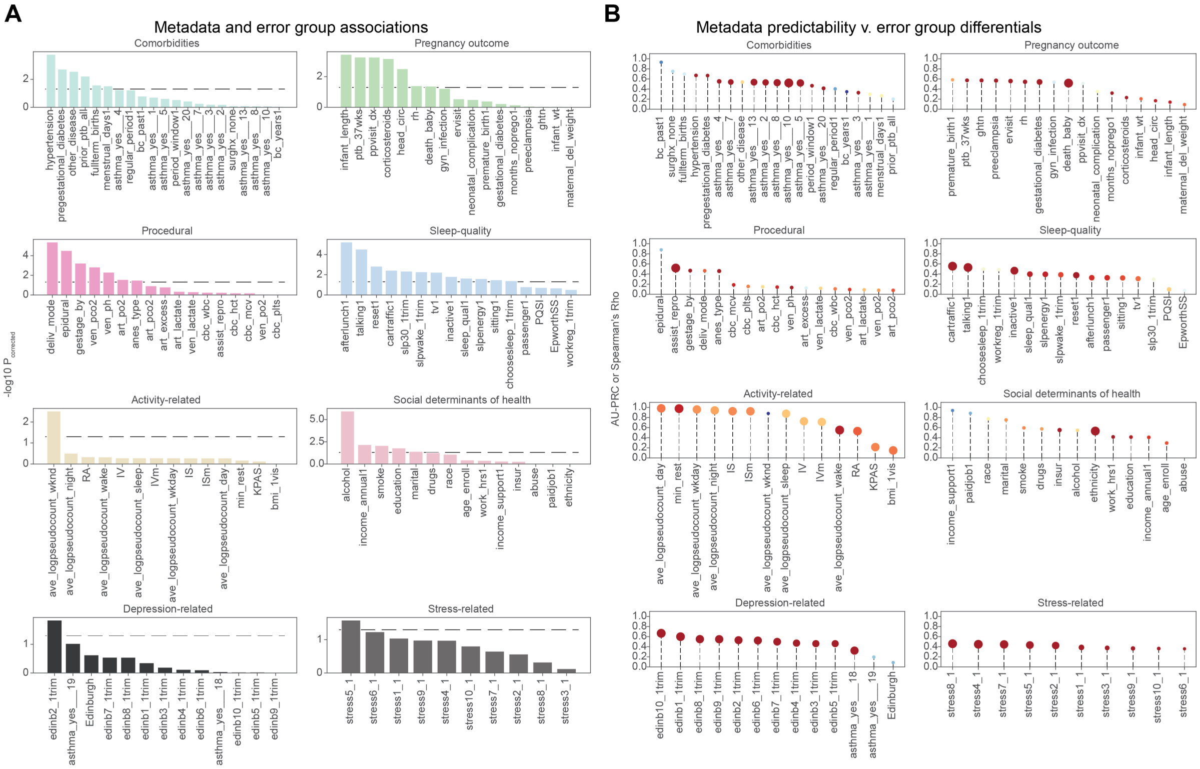

Supplementary Figure 5: Metadata variable categories and differences between error group differences and predictability. (**A**) Differences in identified error groups showing P value corrected for multiple comparisons. Dashed line shows significance threshold $P = 0.05$. (**B**) Predictability, defined as AU-PRC or the absolute value of Spearman's ρ. Color and size of the point represents the percent difference in predictability (AU-PRC or MAPE) between error groups.

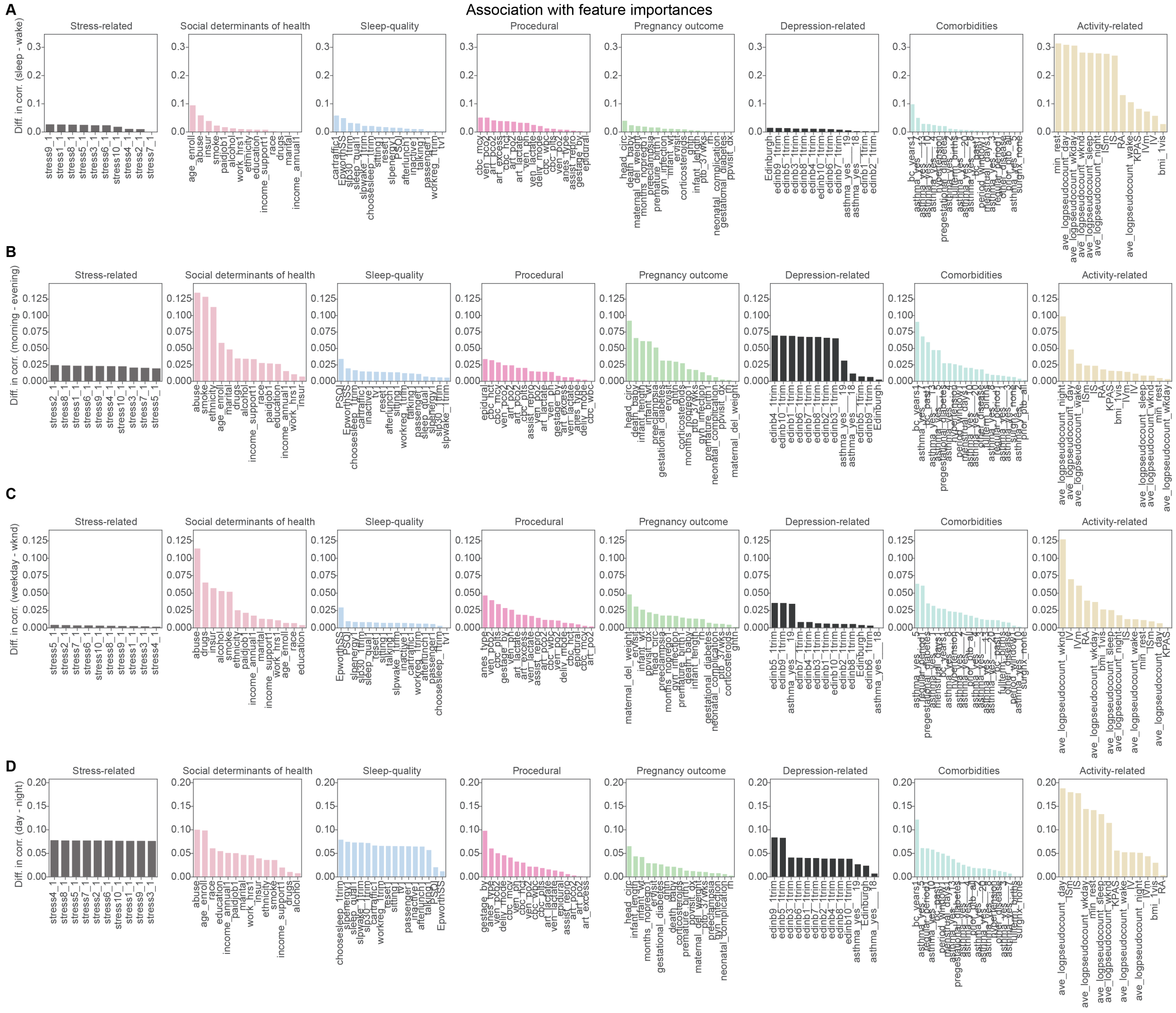

Supplementary Figure 6: Associations of time-of-day or -week variable groups with feature importance scores. (**A** - **D**) Difference between time or period of day or week, as indicated in the y-axis label using a custom association score (see Methods). Feature attribution scores were sampled from each metadata variable group to measure association in accordance with the indexed time for that feature.

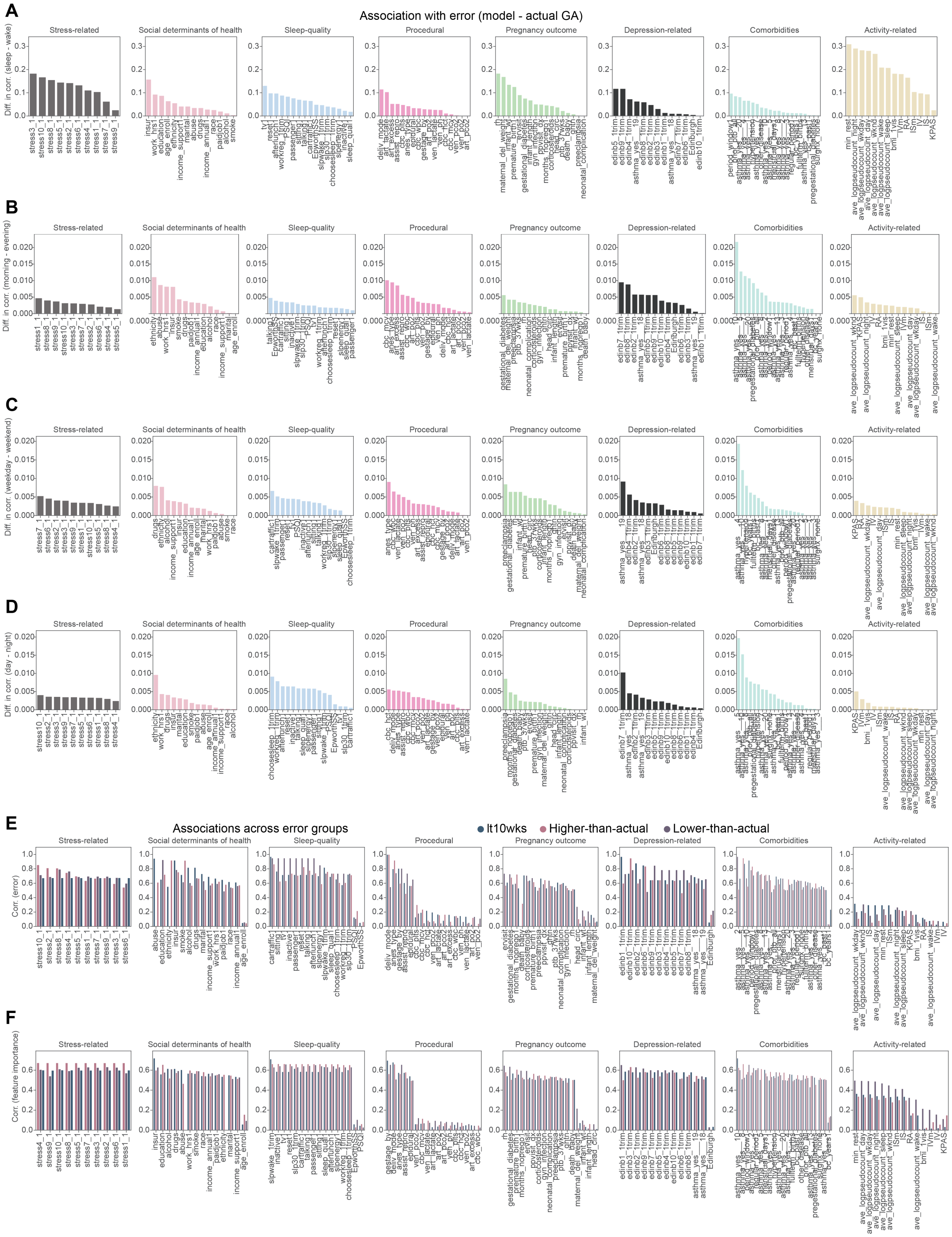

Supplementary Figure 7: Associations of time-of-day or -week variable groups and error groups with top-1 series2signal model error and feature importance. (**A** - **D**) Difference between time or period of day or week, as indicated in the y-axis label using a custom association score (see Methods). (**E**) Association of error groups with feature importance and model error (**F**). Feature attribution scores were sampled from each metadata variable and error group to measure association.

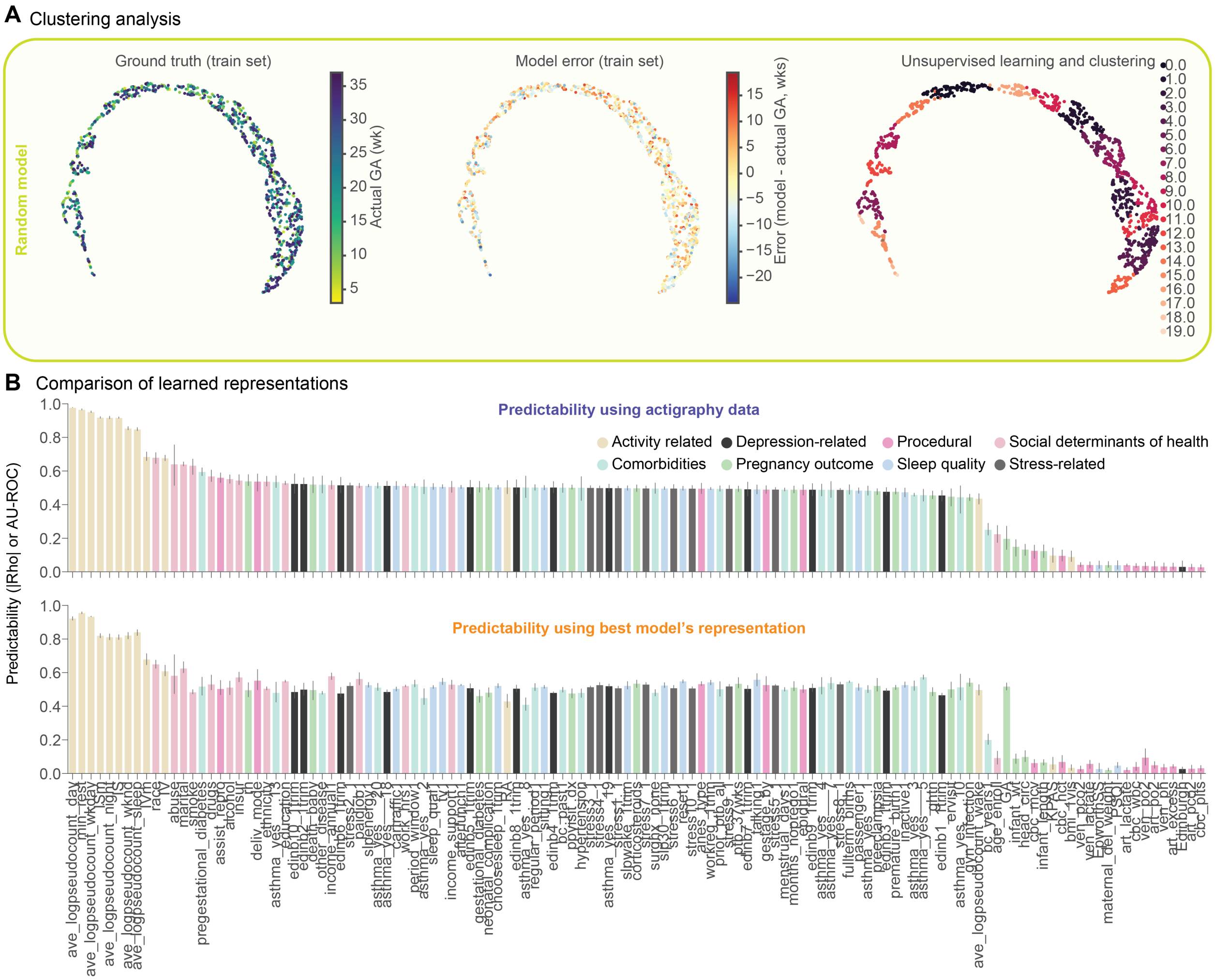

Supplementary Figure 8: series2signal semantic clustering for phenotyping with a random model and predictability of non-linear series2signal embeddings vs. raw input. (**A**) Semantic clustering for series2signal model trained on randomly shuffled GA labels and the associated annotation per cluster. (**B**) Predictability of series2signal's top-1 model learned time-series representation on a kNN classifier or regressor with $k = 5$ (top) versus the log-pseudocount actigrpahy data as input to a lightweight model for the indicated task (bottom).
