# Supplementary material for: Deep representation learning identifies associations between physical activity and sleep patterns during pregnancy and prematurity
Source: NPJ Digit Med. 2023 Sep 28;6:171. doi: 10.1038/s41746-023-00911-x (PMC10539360; doi:10.1038/s41746-023-00911-x)
Supplement: Supplementary file 2 — Reporting Summary [file 41746_2023_911_MOESM2_ESM.pdf]

nature portfolio

Corresponding author(s): Nima Aghaeepour

Last updated by author(s): Jul 6, 2023

Reporting Summary

Nature Portfolio wishes to improve the reproducibility of the work that we publish. This form provides structure for consistency and transparency in reporting. For further information on Nature Portfolio policies, see our Editorial Policies and the Editorial Policy Checklist.

Please do not complete any field with "not applicable" or n/a. Refer to the help text for what text to use if an item is not relevant to your study.
For final submission: please carefully check your responses for accuracy; you will not be able to make changes later.

Statistics

For all statistical analyses, confirm that the following items are present in the figure legend, table legend, main text, or Methods section.

n/a	Confirmed	
☐	☒	The exact sample size (*n*) for each experimental group/condition, given as a discrete number and unit of measurement
☐	☒	A statement on whether measurements were taken from distinct samples or whether the same sample was measured repeatedly
☐	☒	The statistical test(s) used AND whether they are one- or two-sided<br>*Only common tests should be described solely by name; describe more complex techniques in the Methods section.*
☐	☒	A description of all covariates tested
☐	☒	A description of any assumptions or corrections, such as tests of normality and adjustment for multiple comparisons
☐	☒	A full description of the statistical parameters including central tendency (e.g. means) or other basic estimates (e.g. regression coefficient) AND variation (e.g. standard deviation) or associated estimates of uncertainty (e.g. confidence intervals)
☐	☒	For null hypothesis testing, the test statistic (e.g. *F*, *t*, *r*) with confidence intervals, effect sizes, degrees of freedom and *P* value noted<br>*Give P values as exact values whenever suitable.*
☒	☐	For Bayesian analysis, information on the choice of priors and Markov chain Monte Carlo settings
☐	☒	For hierarchical and complex designs, identification of the appropriate level for tests and full reporting of outcomes
☐	☒	Estimates of effect sizes (e.g. Cohen's *d*, Pearson's *r*), indicating how they were calculated

Our web collection on statistics for biologists contains articles on many of the points above.

Software and code

Policy information about availability of computer code

Data collection	To summarize data, if data were normal (by D'Agostino and Pearson's test then mean and standard deviation were shown, otherwise median and inter-quartile range was shown. Categorical variables were assessed for differences across groups by Fisher's exact test and continuous variables were compared across groups by a Kruskal-Wallis test. In head-to-head comparisons for continuous data, either Spearman's ρ or Mann-Whitney U tests were used and categorical, when ordinal and compared with other categorical data, was assessed by the Goodman Kruskal Gamma. Categorical-continuous variables were compared by logistic regression, using the categorical variable as binary or multi-class classification and micro-averaging performance per class after adjusting for class imbalance via SMOTE v0.10.1. χ^2 tests were used to compute observed and expected ratios for the prevalence of various metadata variables. Permutation tests were performed for derived metrics for at least $n=1000$ iterations and after the null distribution was created by random sampling over groups and preserving group ratios, a Mann-Whitney U test was performed to statistically compare the difference between observed and null metrics. All P-values were corrected for multiple comparisons by applying the Bonferonni correction method ($p_{corrected} = p*n_{comparisons}$) where appropriate, unless specified. All statistical analyses were performed with \texttt{scipy} v1.11.1, \texttt{numpy} v1.24, and \texttt{scikit-learn} v1.2.2 in Python v3.8.12.
Data analysis	Our series2signal software is available for use on custom datasets through GitHub at \url{https://github.com/nealgravindra/wearables} under an MIT License. Models can be trained and post-hoc inferences can be generated using our new series2signal approach with existing PyTorch models and either CPU or GPU versions. All analyses and results presented in this paper are also available on our GitHub repo for reproducibility and the code used to generate each figure and table is specified in a reproducibility table within the same GitHub repository (\href{https://github.com/nealgravindra/wearables/blob/main/results/reproducibility.md}{see here}).

For manuscripts utilizing custom algorithms or software that are central to the research but not yet described in published literature, software must be made available to editors and reviewers. We strongly encourage code deposition in a community repository (e.g. GitHub). See the Nature Portfolio guidelines for submitting code & software for further information.

Data

Policy information about availability of data

All manuscripts must include a data availability statement. This statement should provide the following information, where applicable:

- Accession codes, unique identifiers, or web links for publicly available datasets
- A description of any restrictions on data availability
- For clinical datasets or third party data, please ensure that the statement adheres to our policy

Raw wearables data, processed wearables data, and the static analysis source code used in this study are available from <https://nalab.stanford.edu/series2signal-gestational-age-clock-for-pregnancy-monitoring/>.

Research involving human participants, their data, or biological material

Policy information about studies with human participants or human data. See also policy information about sex, gender (identity/presentation), and sexual orientation and race, ethnicity and racism.

Reporting on sex and gender	All study participants included gave written informed consent. Data on gender and sex was not included in this study and it was not reported. Instead, only pregnant study participants are considered, and no further data was reported on patient gender identification.
Reporting on race, ethnicity, or other socially relevant groupings	All study participants included gave written informed consent and all survey responses, which include socioeconomic questionnaires, were self-reported by study participants. Study participants self-identified as white, hispanic, black, or other but this variable was not used as part of the analysis in this study, other than as a summary statistic for reporting. All questionnaires have been validated in previous studies.
Population characteristics	1260 individuals were enrolled if they had a singleton pregnancy ≤ 20-weeks gestation during the study period of January 2017 to January 2020. Inclusion criteria were that the individual (A) planned to deliver at Barnes-Jewish Hospital, (B) were English-speaking, and (C) were at least 18 years old. Exclusion criteria were (a) prior incarceration, (b) conception via \textit{in vitro} fertilization, and (c) diagnosis of major fetal anomaly that affected gestational age at delivery by attending physician.
Recruitment	Patients from the Washington University School of Medicine and local community who were planning pregnancy were recruited to the study. Pregnant individuals were included in this study if they were at least 18 years of age, preparing to conceive, and willing to wear an actigraphy monitor throughout their pregnancy. Patients that had multiple gestations, underwent \textit{in vitro} fertilization, had a uterine anomaly, or had used sedatives were excluded. Patients who signed informed consent documents wore activity monitors throughout their pregnancy until delivery.
Ethics oversight	This study was supported by the NIH (R35GM138353, R01HD105256, P01HD106414, 1R01HL139844, 19PABHI34580007, R61NS114926, R01AG058417, P30AG066515), American Heart Association (19PAB / HI34580007), Burroughs Wellcome Fund (1019816), the March of Dimes, the Robertson Foundation, and the Bill and Melinda Gates Foundation (INV-001734, OPP1113682, INV-003225), and the Alfred E. Mann Family Foundation. Institutional Review Board approval was obtained from the Washington University School of Medicine Human Research Protection Office.

Note that full information on the approval of the study protocol must also be provided in the manuscript.

Field-specific reporting

Please select the one below that is the best fit for your research. If you are not sure, read the appropriate sections before making your selection.

☒ Life sciences ☐ Behavioural & social sciences ☐ Ecological, evolutionary & environmental sciences

For a reference copy of the document with all sections, see nature.com/documents/nr-reporting-summary-flat.pdf

Life sciences study design

All studies must disclose on these points even when the disclosure is negative.

Sample size	1260 individuals were enrolled if they had a singleton pregnancy ≤ 20-weeks gestation during the study period of January 2017 to January 2020. Inclusion criteria were that the individual (A) planned to deliver at Barnes-Jewish Hospital, (B) were English-speaking, and (C) were at least 18 years old.
Data exclusions	Exclusion criteria were (a) prior incarceration, (b) conception via \textit{in vitro} fertilization, and (c) diagnosis of major fetal anomaly that affected gestational age at delivery by attending physician.
Replication	Data splits for all trials were separated by patient so that no measurement from the same patient leaked into different splits. To ensure generalizability and reproducibility, we split our data of 1083 patients into train/test/validation splits of 70/15/15 and ensured that our results held in each split. Furthermore, we performed cross-validation, including k-fold CV, in downstream ML and statistical analyses to ensure reproducibility of reported models

Randomization	Study participants were seen at study visits longitudinally to obtain data and samples in each trimester. All study participants were given validated questionnaires about sleep habits and lifestyle and wore actigraphy devices (Motionwatch8, CamNTech, United Kingdom) continuously (24/7) for two-week time periods immediately following their first, second, and third trimester study visits to assess circadian rhythms longitudinally throughout pregnancy and delivery. All participants in this study received the exposure, as well as incentives and reminders and fully charged, 90d actigraphy devices to ensure full data collection. Retrospective sub-groups were created from these participants based on associated clinical data.
Blinding	Not applicable

Reporting for specific materials, systems and methods

We require information from authors about some types of materials, experimental systems and methods used in many studies. Here, indicate whether each material, system or method listed is relevant to your study. If you are not sure if a list item applies to your research, read the appropriate section before selecting a response.

Materials & experimental systems

n/a	Involved in the study	
☒	☐	Antibodies
☒	☐	Eukaryotic cell lines
☒	☐	Palaeontology and archaeology
☒	☐	Animals and other organisms
☒	☐	Clinical data
☒	☐	Dual use research of concern
☒	☐	Plants
☒	☐	

Methods

n/a	Involved in the study	
☒	☐	ChIP-seq
☒	☐	Flow cytometry
☒	☐	MRI-based neuroimaging

This checklist template is licensed under a Creative Commons Attribution 4.0 International License, which permits use, sharing, adaptation, distribution and reproduction in any medium or format, as long as you give appropriate credit to the original author(s) and the source, provide a link to the Creative Commons license, and indicate if changes were made. The images or other third party material in this article are included in the article's Creative Commons license, unless indicated otherwise in a credit line to the material. If material is not included in the article's Creative Commons license and your intended use is not permitted by statutory regulation or exceeds the permitted use, you will need to obtain permission directly from the copyright holder. To view a copy of this license, visit http://creativecommons.org/licenses/by/4.0/
